# Carbon costs of different pathways for reducing fire hazard in the Sierra Nevada

**DOI:** 10.1002/eap.70111

**Published:** 2025-11-02

**Authors:** Yihong Zhu, Daniel E. Foster, Brandon M. Collins, Scott L. Stephens, Robert A. York, Ariel T. Roughton, Emily E. Y. Moghaddas, John E. Sanders, John J. Battles

**Affiliations:** ^1^ Department of Environmental Science, Policy, and Management University of California, Berkeley Berkeley California USA; ^2^ USDA Forest Service Pacific Southwest Region Vallejo California USA; ^3^ Berkeley Forests University of California, Berkeley Berkeley California USA

**Keywords:** California conifer forest, carbon dynamics, frequent‐fire regime, net ecosystem productivity, prescribed fire, wildfire mitigation

## Abstract

Restoring a low‐intensity, frequent‐fire regime in fire‐prone forests offers a promising natural climate solution. Management interventions that include prescribed fire and/or mechanical treatments have effectively reduced fire hazards in the Western United States, yet concerns remain regarding their impact on forest carbon storage. This study used results from a long‐term, replicated field experiment to assess the impacts of a restored disturbance regime on carbon dynamics in a Sierra Nevada, mixed conifer forest. The carbon consequences of the treatments were compared to a dynamic baseline of untreated controls (Control). After 19 years of wildfire mitigation, all treated stands stored less carbon than Control, but a larger proportion was sequestered in wildfire‐resistant pools (i.e., large trees or fire‐resistant species). Notably, only the most intensive treatment regime—thinning, mastication, and prescribed fire (Mech+Fire)—became a net carbon source by Year 20 (−60 MgC/ha). Annual average net ecosystem productivity (NEP) in Control and prescribed fire‐only (Fire, 5.6–5.8 MgC/ha/year) more than doubled that of the mechanical treatments (2.0–2.1 MgC/ha/year). Moreover, temporal trends diverged. By the 3rd post‐fire interval, the live vegetation carbon accumulation stalled in Control (0.9 ± 1.0 MgC/ha/year, mean ± SE) and accelerated in Fire (6.6 ± 1.2 MgC/ha/year). In contrast, surface fuel recovery was initially faster in Fire but slowed significantly by the 3rd interval, suggesting that the increased productivity under a frequent‐fire regime does not necessarily lead to rapid surface fuel buildup once the regime is established. A simulated wildfire in Year 20 killed 6×–16× more live tree carbon in Control (46% mortality). Still, Control maintained the highest post‐fire carbon storage. Despite the inherent carbon costs of wildfire mitigation, our 20‐year study highlights management pathways that minimize the trade‐off between wildfire hazard and carbon storage in Sierra Nevada mixed conifer forests.

## INTRODUCTION

Critical unknowns in contemporary forest management include the explicit trade‐offs between the forest's potential to mitigate climate change (Walker et al., 2022) and the vulnerability of stored carbon to disturbances (Anderegg et al., 2020). Globally, Mo et al. (2023) estimated that conserving existing forests (e.g., allowing forests to mature and restoring deforested land) could remove 130 PgC from the atmosphere, which represents a significant fraction of the remaining carbon budget (sensu Rogelj et al., 2019) that must be maintained to limit warming to <2°C. Meanwhile, the permanence of forest carbon once stored is threatened by contemporary and likely future disturbances (Seidl et al., 2017).

In their assessment of natural climate solutions for the United States, Fargione et al. (2018) recommended restoring a low‐intensity, frequent‐fire regime to the fire‐prone forests of the Western United States, based on the potential carbon benefit of avoiding large‐scale “tree‐killing” wildfire. However, they acknowledged the uncertainty of their counterfactual analysis and noted the need to better quantify the carbon implications of a frequent‐fire disturbance regime. The impact of reintroducing fire and mimicking fire effects (i.e., fire surrogates, sensu McIver et al., 2009) on the forest carbon balance depends on multiple factors including: the probability of wildfire occurrence, the reduction in wildfire severity (i.e., less overstory tree mortality), the fire vulnerability of carbon pools (e.g., large, fire‐resistant trees), susceptibility to nonfire stressors (e.g., drought, insects, and disease), post‐treatment net ecosystem productivity (NEP), and the longevity of treatment effects (Campbell et al., 2012; Dore et al., 2016; Hurteau & North, 2009; Kalies & Kent, 2016; Pellegrini et al., 2021; Stephens et al., 2009). In addition, the feasibility of implementing such active management at sufficient scale both temporally and spatially is contingent on the economic and social costs (Hartsough et al., 2008; Wunder et al., 2021). There are fixed economic costs associated with planning, labor, equipment usage and maintenance, and transportation to the treatment sites. These expenses vary by site and the scale of treatments (North et al., 2015). Prescribed fire ($62–$1235/ha) in general has a lower cost than mechanical treatments ($618–$6126/ha; Hunter et al., 2007, Hartsough et al., 2008). However, the social costs of prescribed fire, in particular smoke production and the potential for escape, tend to be higher (Brunson, 2023).

Wildfire mitigation and forest restoration treatments that included prescribed fire with or without mechanical fuel reduction have proven effective at reducing high‐severity fire and tree mortality in the Western United States (Davis et al., 2024; Kalies & Kent, 2016). Mechanical fuel reduction involves thinning (i.e., whole‐tree removal) and mastication (e.g., chipping and shredding). Although whole‐tree removal is involved, it is not equivalent to clearcutting or logging. Instead, mechanical thinning emphasizes the removal of smaller trees and shrubs in order to reduce the potential for crown fire (Stephens et al., 2009).

For the Sierran mixed conifer forests, Stephens et al. (2024) demonstrated that both prescribed fire and mechanical fuel reduction treatments can be effective wildfire mitigation strategies. However, all these treatment pathways explicitly reduce carbon stores (Foster et al., 2020). Therefore, quantifying the carbon costs associated with these practices is crucial to management and policy (Peng et al., 2023). This study evaluated the impacts of a restored fire regime on carbon dynamics in a Sierra Nevada, mixed conifer forest. It used the results from a long‐term, replicated field experiment to address the following questions:

1. How much did wildfire mitigation treatments applied over the last two decades reduce carbon stocks?
2. Did the treatments increase net ecosystem productivity compared to the controls (Control, i.e., no active management but continued fire suppression)?
3. For the prescribed fire treatment (Fire), how did repeated entries affect the recovery of carbon pools in the live vegetation and surface fuels?
4. Do the restored forests maintain a larger fraction of their live tree carbon in wildfire‐resistant tree pools?
5. How well do the restored forests mitigate the carbon consequences of a simulated wildfire?

A companion paper (Stephens et al., 2024) focused on the efficacy of the treatments in reducing fire hazard and improving forest resilience. To inform pressing policy questions on the trade‐offs between wildfire mitigation and carbon storage, results from this study were combined with results from the earlier synthesis to quantify the benefits and costs associated with these strategies. Carbon cost was defined as the magnitude of carbon storage (in megagrams of carbon per hectare) that was not realized due to the treatments (Peng et al., 2023). Thus, an integrative sixth question was:

6. What is the net carbon cost of a 20‐year program of wildfire mitigation and forest restoration in a fire‐prone ecosystem?

This question was evaluated from two perspectives: (1) the prevailing condition where the forest did not encounter a wildfire during the last 20 years, and (2) the counterfactual scenario where the forest encountered a wildfire after 19 years of treatments.

## METHODS

### Site description

This experiment was performed at the Blodgett Forest Research Station (Blodgett Forest), approximately 20 km east of Georgetown, California (38°54′45″ N, 120°39′27″ W). The main property of Blodgett Forest is 1780 ha in area; it spans an elevation gradient between 1100 and 1410 m above sea level. The climate is Mediterranean with a summer drought period that extends into the fall (York et al., 2021). Blodgett Forest supports a productive California mixed conifer forest. Species dominance is shared by six tree species (Appendix S1: Table S1): white fir (*Abies concolor*), incense‐cedar (*Calocedrus decurrens*), Douglas‐fir (*Pseudotsuga menziesii*), ponderosa pine (*Pinus ponderosa*), sugar pine (*Pinus lambertiana*), and California black oak (*Quercus kelloggii*). Fire was common at Blodgett Forest before the removal of Indigenous stewardship. Between 1750 and 1900, the median composite fire interval at the 9–15 ha spatial scale was 4.7 years, with a fire interval range of 4–28 years (Stephens & Collins, 2004). See Appendix S1: Section S1 for details.

### Experimental design

Twelve similar experimental units across Blodgett Forest were selected to provide a site‐level replication of the national Fire and Fire Surrogate (FFS) Study (McIver et al., 2009). Three replicates of four treatments were randomly assigned to units. Pre‐treatment measurements confirmed that units were comparable in forest composition and structure (Appendix S1: Tables S1 and S2). The four treatments—control, prescribed fire, mechanical treatments, and mechanical treatments followed by prescribed fire—were initially installed in late 2001 and 2002 (Stephens & Moghaddas, 2005) and were designed to reduce fire severity using management practices common to the northern Sierra Nevada (Agee & Skinner, 2005; Schwilk et al., 2009). See Appendix S1: Section S2 for a more detailed summary on the FFS study.

The experiment used a Before‐After‐Control‐Impact (BACI) design (Stewart‐Oaten & Bence, 2001). This design explicitly controls for pre‐impact differences among the units. This design provides statistical power and robustness on par with randomized controlled trials (Christie et al., 2019). The specifics of each treatment are outlined below; more details are available in Stephens and Moghaddas (2005) and Stephens et al. (2009).

**Control units** (Control) received no treatment during the study period. Wildfires were successfully excluded for the interval reported in this study (2001–2020). Note that while the 2022 Mosquito Fire burned through one of the three control units, our results pre‐date this event.

**Fire‐only units** (Fire) were burned with no mechanical pre‐treatment using strip head fires three times. The 1st entry was in Fall 2002; the 2nd entry was in Fall 2009; and the 3rd entry was Fall 2017. Burn plans prescribed the following weather parameters: air temperature between 0°C and 10°C; relative humidity >35%, and wind speed <5 km/h. The desired 10‐h fuel stick moisture content was 7%–10%.

**Mechanical‐only treatment units** (Mech) experienced two treatments, both of which include a two‐stage prescription. The first one was in 2001. Units were first crown thinned and then thinned from below to remove ladder fuels. Thinning guidelines emphasized retaining large trees in an even mix of conifer species in the post‐treatment forest (Stephens & Moghaddas, 2005). Individual trees were felled, bucked, and limbed using a chainsaw, and boles were then yarded to landings with ground‐based skidders. The sawlogs were transported to a sawmill; tops and limbs were masticated and left to decompose. Generally, residual trees were well spaced with little overlap of live crowns of the dominant and co‐dominant trees. The thinning step retained 28–34 m^2^/ha of basal area, with larger diameter trees contributing most of the remaining basal area.

Following the harvest, approximately 90% of understory conifers and hardwoods up to 25 cm diameter at breast height (breast height = 1.37 m, dbh) were masticated in place using an excavator‐mounted rotary masticator. A 2nd Mech‐only treatment was done with understory mastication in 2017 followed by a second thinning from below in 2019. This 2nd thinning operation incorporated mechanized harvesting and yarded whole trees to landings, thus leaving less of the harvest‐related fuels (“activity fuels”) in the units. In both operations, treatments were specifically designed to reduce tree density while also reducing surface fuels. Treatment impacts on surface fuels included limiting fuel input over time (via harvest of trees that would have died from competition), removing ladder fuels (via mastication), and speeding up the decomposition of surface fuels (also via mastication).

**Mechanical+Fire units** (Mech+Fire) underwent the same initial treatment as Mech in 2001, but following mastication, they were broadcast burned using a backing fire in the fall of 2002. The fire consumed large amounts of activity fuel that came from the mastication of medium‐sized trees and harvesting slash. In 2017, shrubs and small trees that developed following 2002 were masticated a second time (similar to Mech), but larger trees were not thinned given their low levels of stocking. The units were then burned with strip head fires in 2018. The fire consumed the activity fuel that came from the mastication of shrubs and small trees. After this 2nd fire, salvage harvesting of clumps of fire‐killed trees occurred in 2019 in Mech+Fire. The two harvest operations in Mech+Fire followed the same procedures as in Mech.

**Wildfire simulations** were applied to each unit with inputs based on the 2020 forest and fuel conditions. Model implementation replicated the procedures used in Stephens et al. (2024). Fuel model inputs and the target indices were derived using a weighted average from the two “best” fuel model assignments given the measured fuel conditions. Weather for each simulation was under “severe” conditions: wind speed = 32 km h^−1^, air temperature = 21°C, 1‐h fuel moisture = 3%, 10‐h fuel moisture = 4%, 100‐h fuel moisture = 5%, 1000‐h fuel moisture = 10%, duff fuel moisture = 15%, and live fuel moisture (woody and herb) = 70%.

### Data collection

In each experimental unit, 20 permanent 0.04‐ha circular plots were established on a 60‐m grid initiated with a random starting point. Plot locations were restricted to a 10‐ha core area in the center of each unit to avoid edge effects (Stephens & Moghaddas, 2005). To quantify responses to the treatment regime, the sampling design prioritized pre‐ and post‐treatment measurements. Specifically, comprehensive field inventories were conducted in all units in the summers of 2001, 2003, 2009, 2016, and 2020 (Appendix S1: Table S3). In addition, immediately after the prescribed fire treatments in 2009 and 2016, “update” inventories were conducted in 2010 (2nd entry) and 2017 (3rd entry). Forest carbon pools were organized for sampling and reporting into the following categories: aboveground live tree, aboveground standing dead tree (snag), aboveground understory, fine woody debris (1‐, 10‐, and 100‐h timelag fuel classes), coarse woody debris (1000‐h and greater timelag fuel classes), litter, duff, and soil organic carbon (SOC) in the 0‐ to 15‐cm layer of mineral soil. Outlined below are the core sampling protocols for each carbon pool with modifications noted for specific inventories.

#### Live tree

For the comprehensive inventories, all live trees ≥11.4 cm dbh in the 0.04‐ha plot were tagged and identified to species. For live trees, dbh, total height, height to live crown base, and crown position (dominant, co‐dominant, intermediate, and suppressed) were recorded. Smaller live trees (1.0 cm ≤ dbh <11.4 cm) were sampled in 0.004‐ha subplots in 2001, 2003, 2009, and 2016. In 2001, 2003, and 2009, species, dbh, total height, and height to live crown base were recorded for all smaller live trees. In 2016, small trees were tallied by species and binned by 2.54‐cm‐wide‐diameter classes. Each tallied small tree was assigned a dbh within its bin range from a uniform distribution and assigned a total height based on observed relationships between dbh and height for small trees. In 2020, sampling of smaller trees was expanded to the entire 0.04‐ha plot; dbh and height were measured along with species identification. For the post‐fire update inventories of 2010 and 2017, the status of the tagged live trees post‐fire was assessed, and any changes were recorded. Trees not impacted by the fire were assigned the pre‐treatment size given the one‐year difference between pre‐ and post‐treatment measurements.

#### Snag

All snags ≥11.4 cm dbh were tagged, identified to species if possible, and measured for dbh and total height (2001, 2003, and 2009 inventories). In 2016, only snags ≥20.5 cm dbh were recorded, but in addition to dbh and total height, decay state was assessed. In 2020, all snags ≥11.4 cm dbh were measured and assigned a decay class (United States Forest Service, 2024). For the post‐fire update inventories of 2010 and 2017, the status of snags post‐fire was assessed, and any changes were recorded. Snags not impacted by the fire were assigned the pre‐treatment measurements.

#### Understory

The 0.04‐ha plot was searched to identify all shrubs and herbs (i.e., forbs and grasses) present. Percent cover was visually estimated by species. Cover estimates were binned into classes of <5%, 5%–25%, and 25%–100%. The bins were interpreted as central values of 2.5%, 15%, and 63%, respectively, and these percent‐cover categories were used to estimate the total area of cover by each species on each plot. In the 2016 and later inventories, the average height of each shrub species present was estimated along with the cover class.

#### Fine and coarse woody debris

Surface and ground fuels (coarse woody debris, fine woody debris, litter, and duff) were sampled along radial transects (11.3 m) at two random azimuths in each 0.4‐ha plot using the line intercept method (Brown, 1974). Fuel measurements were taken on the same schedule as the vegetation measurements. The 1‐h (0–0.64 cm) and 10‐h (0.64–2.54 cm) fuels were sampled from 0 to 2 m, 100 h (2.54–7.62 cm) fuels from 0 to 3 m, and 1000‐h (>7.62 cm) and larger fuels from 0 to 11.3 m on each transect. Surface and ground fuels were assessed in all inventories using the same protocol.

#### Duff and litter depth

Litter and duff depths were measured at 0.3 and 0.9 m on each fuel transect. Per convention, litter was defined as the Oi fraction of the forest floor; duff included the Oe and Oa fractions (Brown, 1974).

#### Soil

Soil samples were collected in 2001, 2003, 2016, and 2020 with one exception. The most recent soil samples in Mech were collected in 2017 after the 2nd mastication application but before the understory thinning. In 2001 and 2003, six soil samples were obtained per plot. Litter and duff were collected in a 15‐cm × 15‐cm rectangular block; mineral soil to 15 cm depth was collected with a core (Moghaddas & Stephens, 2007). Litter, duff, and mineral soil samples from each plot were aggregated prior to analysis. In 2016, two or three random samples were collected in each plot. Litter and duff were collected with a 30‐cm‐diameter core; mineral soil to a 15‐cm depth was collected with a 5‐cm cylindrical core. Litter and duff were combined for processing. Each subsample (litter+duff and 0–15‐cm mineral soil layer) was analyzed separately. In 2020 (2017 in Mech), one sample was collected per plot. Litter and duff were collected in a 30‐cm‐diameter core; two mineral soil cores (5‐cm diameter) were collected at 0–15 cm and 15–30 cm depth. Litter and duff samples were combined for processing; the mineral soil samples were processed separately. In all cases, the depths of the litter layer and litter+duff were measured in the field.

### Estimating carbon pools and fluxes

Given the plethora of terms used to describe carbon dynamics across disciplines, the definitions for terms used in this study are provided in Table 1. The methods used to estimate carbon pools and fluxes are outlined below. For details, see the corresponding subsections in Appendix S1: Section S5.

**TABLE 1:** Brief definition of the terms used to describe carbon pools and fluxes in this study.

Term	Definition	Reference
Total measured carbon	Total measured carbon is the sum of all carbon pools measured in the field.	Appendix S1: Section S3.1
Total net ecosystem carbon balance (NECB)	Change in total measured carbon between 2001 and 2020.	Appendix S1: Section S3.2
Net ecosystem productivity (NEP)	NECB minus carbon emitted by prescribed fire and wood removals during harvest operations.	Appendix S1: Section S3.3
Live vegetation recovery/accumulation	Annual accumulation rate of carbon in the aboveground live vegetation (tree, shrub, and herb).	Appendix S1: Section S3.4
Surface fuel recovery/accumulation	Annual accumulation rate of carbon in the fine surface fuel (litter, 1‐, 10‐, and 100‐h fuels).	Appendix S1: Section S3.5
Wildfire‐resistant carbon	Aboveground live tree carbon stored in large trees (dbh ≥ 76.2 cm) and more wildfire‐resistant species (ponderosa pine and sugar pine).	Appendix S1: Section S3.6
Fire emissions	Carbon emitted during burning operations.	Appendix S1: Section S3.7
Long‐lived wood product (LLP)	Carbon stored in durable wood products from harvested trees.	Appendix S1: Section S3.8
Wood removals	Carbon removed from the forest during harvest operations.	Appendix S1: Section S3.9
Carbon cost	The magnitude of carbon sequestration that was not realized due to the treatments with the results expressed in terms of mitigation impact in 2001.	Appendix S1: Sections S3.10 and S8; Table S5
Stable carbon	Aboveground live tree carbon stored in large fire‐resistant trees (ponderosa pine and sugar pine with dbh ≥ 72.6 cm) that are expected to survive after a wildfire.	Appendix S1: Section S3.11

#### Aboveground live tree

Aboveground live tree biomass was calculated from tree measurements (species, dbh, and height) by using regional biomass equations (Forest Inventory and Analysis, 2010) to predict wood biomass (stem, bark, and branches) and using Jenkins' ratios (Jenkins et al., 2003) to estimate foliage biomass. Aboveground live tree biomass is the sum of the stem, bark, branch, and foliage mass. The biomass estimate was converted to carbon using a carbon:biomass ratio of 0.48 (IPCC, 2003). These estimates were summed and scaled by plot size to estimate the aboveground live tree carbon pool (in megagrams of carbon per hectare).

#### Snag

Snag biomass was initially estimated using the wood and bark equations for live trees described above and then reduced using a live‐to‐dead biomass ratio of 0.88, which is the live‐to‐dead ratio for all Sierra Nevada mixed conifers in decay class 2 (Cousins et al., 2015; Foster et al., 2020). The calculated biomass was converted to carbon using a carbon fraction of 0.51 for snags in decay class 2 (Cousins et al., 2015). These estimates were summed and scaled by plot size to estimate the snag carbon pool (in megagrams of carbon per hectare).

This simple discounting of snag carbon was necessary because decay class based on the FIA protocol (United States Forest Service, 2024) was not systematically recorded for snags until 2016. However, for a subset of the snags in pre‐treatment (2001) inventory, wildlife habitat assessments were available, which recorded variables (e.g., limb condition, wood hardness, and top presence) that were crosswalked to the comparable FIA decay class (Foster et al., 2020). The modal decay class for snags was 2 in both the pre‐treatment and the 2016 inventories. For consistency across the two inventories, the same biomass decay and carbon fraction ratios were applied to all snags. Admittedly, this assumption likely overestimates the biomass of old snags that have experienced significant degradation and decay. However, snag biomass is a relatively small pool, and this bias does not affect the direction or magnitude of the cost of carbon analyses.

#### Understory

To estimate shrub biomass, shrub cover was converted into the estimated number of average‐sized individuals present based on allometric results (McGinnis et al., 2010). The per‐individual biomass was calculated using the species‐specific allometric equations that predict total shrub biomass as a function of crown diameter and height (McGinnis et al., 2010). These individual biomass estimates were summed and scaled by plot size to get shrub biomass on each plot (in megagrams per hectare) and then converted to carbon (in megagrams of carbon per hectare) using a carbon:biomass ratio of 0.49 (Chojnacky & Milton, 2008). The carbon density of herbs was calculated from cover using methods described in Campbell et al. (2009). The understory carbon pool was estimated by summing the shrub and herb carbon pools.

#### Fine and coarse woody debris

The biomass of woody debris was estimated from the transect data using equations and species‐specific coefficients for Sierra Nevada forests (Van Wagdendonk et al., 1996; Van Wagtendonk et al., 1998). The coefficients used for each plot were generated by calculating the weighted mean of the species‐specific coefficients, with the weights derived from the relative basal area of the tree species present (Stephens, 2001). The two transect‐level estimates for woody debris biomass on each plot were averaged to generate a plot‐level estimate, which was converted to megagrams of carbon per hectare by assuming a carbon:biomass ratio of 0.5 for coarse and fine woody debris (IPCC, 2003).

#### Litter and duff

Litter and duff samples were oven‐dried at 65°C to a constant weight and then ground through a 1‐mm sieve. A 10‐g sample of the 1‐mm fraction was ground in a ball mill to pass a 60‐mesh screen and then analyzed for total carbon by combustion (Moghaddas & Stephens, 2007). Results from the 2016 analyses were used to quantify the consistency in the carbon fraction in litter and duff as estimated by depth. At the plot level, litter and duff depths were reliable predictors of biomass (*R* ^2^ = 0.85, *N* = 267). Moreover, the carbon fraction of litter and duff samples demonstrated very little variability (Appendix S1: Table S4). Therefore, to estimate carbon in the litter and duff pools, the depth measurements taken in the fuel transects were used to calculate biomass density based on methods outlined in Moghaddas and Stephens (2007). Litter biomass density was converted to carbon density using a carbon:biomass ratio of 0.463; for duff, biomass was converted to carbon using a ratio of 0.362 (Appendix S1: Table S4).

#### Mineral soil carbon

To determine the carbon content in the mineral soil, air‐dry soil samples were sieved to obtain the fine fraction (particle size <2 mm in diameter). A subsample of the fine fraction was dried to constant mass at 105°C. A 10‐g sample of the fine fraction was ground in a ball mill to pass a 60‐mesh screen and then analyzed for total carbon by combustion (Moghaddas & Stephens, 2007). Soil bulk density (in grams per cubic centimeter) was determined based on the fine‐fraction mass and total volume of each soil core. The bulk density of each sample and the carbon content of the fine fraction were used to determine surface soil carbon on a per‐hectare basis (in megagrams of carbon per hectare).

#### Fire emissions

The First‐Order Fire Effects Model (FOFEM 6.7, Keane & Lutes, 2018) was applied to estimate atmospheric carbon emissions (e.g., CO_2_, CO, and CH_4_) from both the prescribed fire (using Rx emission factors) and the simulated wildfire (using WF emission factors). The total emitted carbon was calculated based on the mass percentage of carbon compounds in the emission estimates. For the prescribed fires, FOFEM was parameterized with the prevailing weather conditions at the time of the burn, the measured pre‐fire fuel loads, and the measured post‐fire tree mortality. For the simulated wildfires, FOFEM used the weather conditions in the wildfire simulation, the measured 2020 (pre‐wildfire) fuel loads, and the simulated post‐fire tree mortality (Kennedy et al., 2020). Recent research at Blodgett Forest documented the skill of FOFEM in predicting emissions with measured pre‐fire fuel loads (Tasnia et al., 2025).

#### Wood product carbon

Fates of the harvested woody material vary by product types (IPCC, 2006). Harvested softwood sawlogs in this study were used for dimensional lumber and nonstructural products. Since these uses met the standards for long‐lived products (LLP, Skog, 2008), these products were counted as a stable carbon stock for the 2001–2020 period. Harvested hardwood sawlogs used for fuelwood were considered as an immediate emission in the year of harvest (Peng et al., 2023). Harvested stems too small for sawlogs (i.e., non‐merchantable) were left on site and processed according to the treatment strategy (i.e., either masticated or masticated and burned).

To calculate the carbon stored in LLP or used for fuelwood, the volume of all harvested sawlogs was estimated with regional volume equations based on dbh and height (Forest Inventory and Analysis, 2010). Sawlog volume for LLP only included conifers larger than 22.9 cm dbh (from a 0.3‐m stump to a 15.2‐cm‐diameter top). Based on recent estimates of California mill efficiency (Buchholz et al., 2021), 67.6% of the sawlog volume contributed to LLP. To account for the fossil fuel emissions associated with harvest and transport, 5% of carbon stored in LLP was deducted from this pool (Buchholz et al., 2021). The kerf and trimmings from the processing (i.e., 22.4% of sawlog volume) are typically used as energy feedstock and thus were counted as an emission (Buchholz et al., 2021). Sawlog volume for fuelwood includes hardwoods larger than 27.9 cm dbh (from a 0.3‐m stump to 20.3‐cm‐diameter top, Forest Inventory Analysis, 2010). These volumes were converted to carbon using the same methods to estimate live tree carbon, namely biomass was estimated from stem volume using species‐specific wood densities and then multiplied by a carbon:biomass ratio of 0.48.

In the 1st Mech treatment (2001), the tops and limbs were left in the forest; in the 2nd entry, the tops and limbs were hauled to the landing and burned. Thus, carbon in tops and limbs was added to the downed woody debris pools in 2001 but was an emission in 2019.

#### Carbon cost

The cost of per‐unit carbon emission is equivalent to the cost of per‐unit mitigation. The mitigation cost is expected to decline with time due to innovation in mitigation technologies. This expectation of declining costs is based on two assumptions. One, existing mitigation options will get cheaper (Pacala & Socolow, 2004). For example, the cost of solar panels and electric vehicles has dropped sharply (Gillingham & Stock, 2018). Two, the development of novel technologies will make carbon capture more efficient and more cost‐effective (Wilberforce et al., 2021). In addition, the discount rate explicitly assigns a higher value to earlier mitigation efforts. It is an explicit recognition of the cumulative impact of increasing emissions on global warming. This analysis followed previous studies (Searchinger et al., 2018) and applied a discount rate of 4% per year. The results were reported in 2001 units to reflect “start‐year equivalent” costs.

The annual carbon cost of treatments was based on the difference in the annual flux between Control and wildfire‐mitigation treatments (Peng et al., 2023). Any reduction in annual flux compared to Control was considered an emission; any increase, a sequestration. Emissions or sequestrations were assigned to the year the treatment was applied. The timing of the treatments varied over the course of the study, with measurements bracketing each intervention (i.e., before and after treatment). To calculate the annual differences in carbon flux, measured changes in carbon storage between two inventories were linearly interpolated. For example, the observed increase in total measured carbon between 2003 and 2009 in Control was allocated to annual increments by dividing by the time between inventories (6 years, Appendix S1: Table S5). Note that in this framework, the fraction of sawlog carbon stored in LLP was counted as a sequestration (i.e., not a cost even though they were no longer on site). In summary, the annual, time‐discounted carbon costs were calculated, and then these annual values were summed across 19 years to obtain the total carbon cost (calculation example in Appendix S1: Table S5).

### Analytical framework

This study leveraged a long‐term experiment with a robust statistical design. It used a stock‐change approach to quantify carbon dynamics over time. There is great value in having repeated observations over 19 years to track stocks and fluxes, but the longevity of the study did pose analytical challenges. A particular challenge was the fact that not every plot was measured in every inventory. Some plots were lost mid‐study to disturbance; other plots were inadvertently skipped during an inventory. Using a different set of plots through time to calculate the carbon balance risks confounding treatment effects with sampling effects. To limit this risk, results were based only on plots measured at every interval. Thus, the sample size reported in the results reflects the number of consistently sampled plots. Another challenge, as noted above (see Data collection), was that data collection protocols changed over time to accommodate resource constraints or to improve assessments. To the extent possible, these inconsistencies were minimized by the procedures for calculating carbon pools (see Estimating carbon pools and fluxes). All results were summarized at the plot level. Statistical analyses were conducted in R version 4.4.0 (R Core Team, 2024).

Generalized linear mixed‐effects models (GLMMs) were used to test the BACI null hypothesis that the observed impacts of the treatment were not different from the after‐treatment trajectory of responses in the controls (Christie et al., 2019). Specifically, the GLMMs examined the interaction between time and treatment on the carbon balance. In all the models, Control was set as the baseline for comparison. Random effects of the unit were always included unless it reported a “singular fit” warning—an indication that the random effect variance was near zero.

### Analysis of treatment effects

Continuous response variables (e.g., total measured carbon) were evaluated using a linear mixed‐effects model (LMM) with a Gaussian distribution. Response variables were transformed when necessary to meet parametric assumptions. For proportional response variables (e.g., the proportion of species‐specific fire‐resistant carbon, proportion of large tree carbon), the default was GLMM with a Beta distribution (Douma & Weedon, 2019). To meet the Beta distribution requirement, proportions equal to 1 in the data records were replaced with 0.99999. Validation tests on zero‐inflation and dispersion provided by the DHARMa package for R (Hartig & Lohse, 2022) were used to decide if including zero‐inflation or dispersion terms was necessary. If a zero‐inflation or dispersion term was required, its structure was based on the model with the lowest Akaike information criterion (AIC) values. Final models passed all validation tests.

To test whether the rates of live vegetation and surface fuel recovery after each entry of fire in Fire were significantly different from Control, another series of LMMs were applied. The treatment type (Fire or Control) was the fixed effect. Pre‐fire live vegetation carbon and disturbance intensity (carbon change during the treatment period) were included as covariates. Unit was included as the random effect. Though the final data contained statistical outliers, they were ecologically reasonable. Thus, they were not removed. Under such minor violations of assumptions, the results of LMMs are found to be generally robust (Schielzeth et al., 2020).

All the LMMs were fitted with the lme4 package (Bates et al., 2024), and all the GLMMs were fitted with the glmmTMB package (Brooks et al., 2024). Statistical results were reported only for the final models. For each model, we defined significant effects as those with a probability of occurring by chance that was less than 0.05.

### Analysis of simulated wildfire

The Forest Vegetation Simulator‐Fire and Fuels Extension (FVS‐FFE, Rebain, 2015) was used to understand the carbon consequence of a wildfire simulated under severe weather conditions after 19 years of treatments (see *Experimental design*). Key fire hazard metrics obtained from the potential fire report included the probability of torching (P‐torch) and potential mortality (P‐mort). P‐torch is a stand‐level index that estimates the proportion of the stand where torching is likely to occur; P‐mort estimates the proportion of tree basal area killed by the fire (Rebain, 2015). Species‐ and size‐specific estimates of the trees killed by the fire, simulated results provided in the FVS‐FFE mortality report, were used to estimate the wildfire impacts on live tree carbon including losses in large‐tree carbon and wildfire‐resistant carbon.

## RESULTS

### Carbon stocks and net ecosystem productivity

**FIGURE 1:**
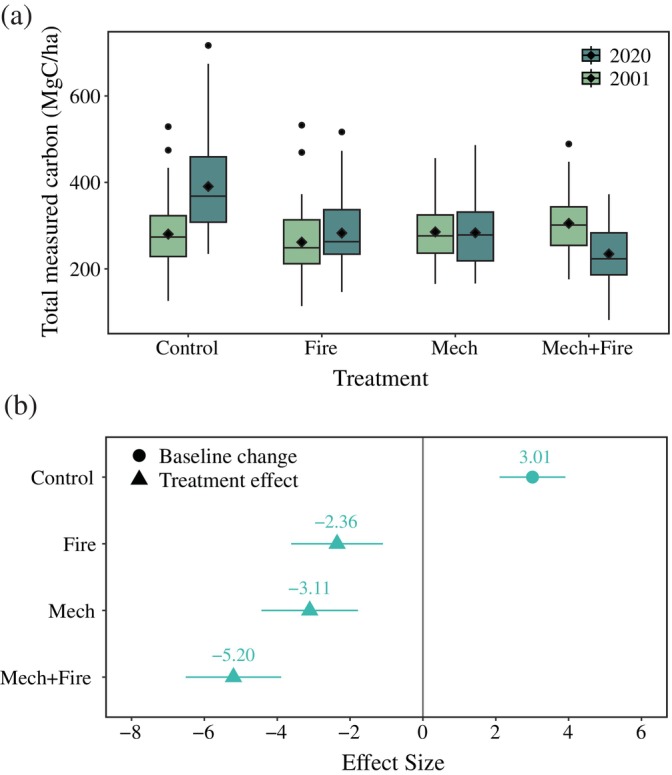
Impact of wildfire mitigation regimes on the carbon stocks (in megagrams of carbon per hectare) at Blodgett Forest Research Station. (a) Change in total measured carbon from 2001 (before treatment) to 2020 (after treatment). The black diamond indicates the plot‐level mean; the midline indicates the median value; the bottom and top of the box indicate the 25th and 75th percentiles, respectively; the vertical lines indicate the 1.5× the interquartile range; and the dots beyond the vertical lines indicate outlying values. (b) The effect size of the interaction term between treatment and time. Control represents the baseline change in total measured carbon from 2001 to 2020. Fire, Mech, and Mech+Fire represent the difference between each treatment's change and the baseline change in Control. The vertical line represents no difference. Full model results can be found in Appendix S1: Table S7.

All wildfire mitigation treatments experienced significant declines in carbon stocks relative to Control (Figure 1). From 2001 to 2020, the carbon stocks in Control significantly increased by 39% (Figure 1, Appendix S1: Tables S6 and S7). In absolute terms, all but two carbon pools (exceptions: shrub and coarse wood debris) increased over time, with the greatest increase (73.4 MgC/ha) in aboveground live trees (Appendix S1: Table S6). In comparison, carbon stock change during the same interval ranged from a 19.6 MgC/ha gain in the Fire units to a 70.7 MgC/ha loss in Mech+Fire (Figure 1; Table 2).

**TABLE 2:** Impact of wildfire mitigation regimes on carbon dynamics at Blodgett Forest Research Station.

Treatment	*N*	Total net ecosystem carbon balance (MgC/ha)		Total fire emission (MgC/ha)		Total wood removals (MgC/ha)		Total net ecosystem productivity (MgC/ha)		Annual net ecosystem productivity (MgC/ha/year)	
		Mean	SE	Mean	SE	Mean	SE	Mean	SE	Mean	SE
Control	53	110.2	8.9	0	…	0	…	110.2	8.9	5.8	0.5
Fire	55	19.6	8.0	−87.1	5.6	0	…	106.7	7.6	5.6	0.4
Mech	46	−2.1	9.6	0	…	−41.8	5.8	39.7	9.2	2.1	0.5
Mech+Fire	48	−70.7	11.6	−87.6	5.1	−21.9	4.3	38.8	8.7	2.0	0.5

*Note*: Total net ecosystem carbon balance = total measured carbon in 2020 − total measured carbon in 2001. Total fire emission refers to emissions during prescribed fire and was estimated by FOFEM. Total wood removals include sawlogs milled to LLP, wood harvests used for fuel, and the offsite (i.e., at the landing) disposal of harvest debris. Total net ecosystem productivity = total net ecosystem carbon balance − total fire emission − total wood removals.

Abbreviations: *N*, number of plots; SE, standard error of the mean.

The carbon stock decrease in Mech+Fire (Figure 1) was due to large carbon removals (109.5 MgC/ha from fire and harvest) that were not offset by NEP (Table 2). While annual NEP in Mech+Fire was comparable to Mech, the Mech+Fire removals were more than double Mech (Table 2). In contrast, the smallest decrease in carbon stocks was observed in the Fire units. While Fire had losses equivalent to 80% of Mech+Fire, annual NEP in the Fire units was almost three times greater than in Mech+Fire (Table 2).

### Recovery and accumulation rates: Live vegetation and surface fuels

**FIGURE 2:**
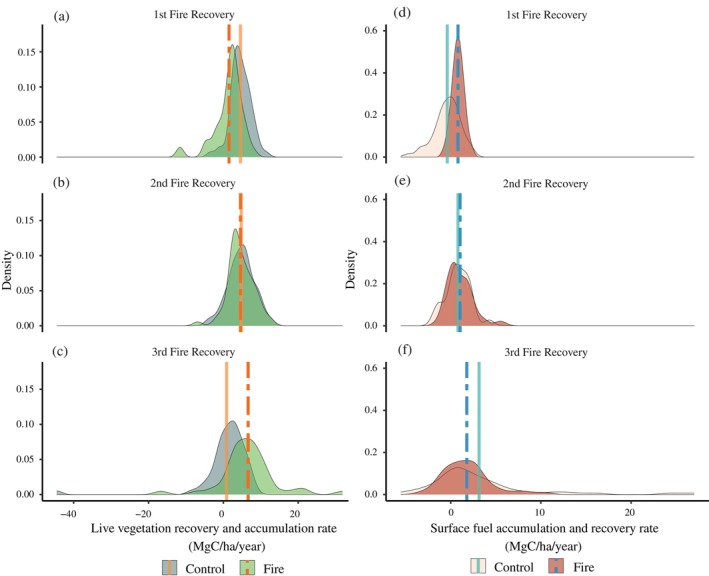
Post‐fire recovery and accumulation rates of live vegetation carbon and surface fuel carbon in the Fire and Control units (in megagrams of carbon per hectare per year) at Blodgett Forest Research Station. The prescribed fire treatments were conducted in 2002, 2009, and 2017. Each recovery period began immediately after a fire and continued until the next fire—1st recovery period: 2003–2009, 2nd: 2010–2016, 3rd: 2017–2020. These recovery periods in Fire correspond to the following accumulation periods in Control—1st accumulation period: 2003–2009, 2nd: 2009–2016, 3rd: 2016–2020. (a–c) Density distribution of recovery and accumulation rates of live vegetation carbon in three periods. (d–f) Density distribution of recovery and accumulation rates of surface fuel carbon in three periods. The vertical solid line represents the mean of Control, and the dashed line represents the mean of Fire. Full model results can be found in Appendix S1: Tables S8 and S9.

Although the average annual NEP was similar in the Control and Fire units (Table 2), the trends in the response of aboveground live vegetation recovery are following contrasting trajectories (Figure 2a–c). In the interval following the 1st prescribed fire (2003–2009), the carbon accumulation rate in live vegetation in Control was significantly faster than in Fire (Figure 2a, Appendix S1: Table S8). During the 1st post‐fire interval, the rate of accumulation in Control was 4.6 ± 0.4 MgC/ha/year (mean ± standard error). This rate of accumulation was more than twice the rate of recovery in the Fire units (1.5 ± 0.5 MgC/ha/year). However, by the 3rd prescribed fire (Figure 2c, interval = 2017–2020), carbon recovery of live vegetation had sped up to 6.6 ± 1.2 MgC/ha/year while carbon accumulation in Control had stalled to 0.9 ± 1.0 MgC/ha/year (effectively 0). In both statistical and ecological terms, this 7× difference in the live vegetation growth was significant (Appendix S1: Table S8).

Interestingly, the pattern of surface fuel recovery between Control and Fire followed the opposite trend (Figure 2d–f). Initially, surface fuel recovery was faster in Fire (Figure 2d). But by the 3rd prescribed fire, the rate of surface fuel accumulation in Control was significantly faster (Figure 2f, Appendix S1: Table S9). Although the rate of fuel recovery increased following each prescribed fire—1st entry = 0.8 ± 0.1 MgC/ha/year 2nd entry = 1.0 ± 0.2 MgC/ha/year; 3rd entry = 1.8 ± 0.4 MgC/ha/year—the surface fuel accumulation rate in Control outpaced these increases. Indeed, surface fuel accumulation rates in Control doubled after each interval: 1st entry = −0.4 ± 0.2 MgC/ha/year; 2nd entry = 0.8 ± 0.2 MgC/ha/year; 3rd entry = 3.1 ± 0.8 MgC/ha/year.

### Wildfire‐resistant carbon

**FIGURE 3:**
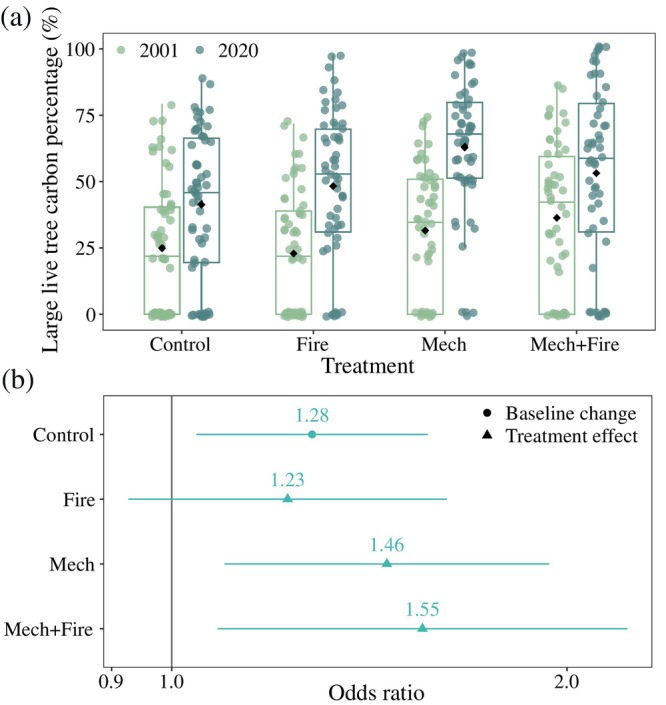
Impact of wildfire mitigation regimes on the large live tree carbon percentage (%) at Blodgett Forest Research Station. Large live tree refers to live tree with a dbh larger than 72.6 cm. (a) Distribution of large live tree carbon percentage in 2001 (before treatment) and 2020 (after treatment). The black diamond indicates the plot‐level mean; the midline indicates the median value; the bottom and top of the box indicate the 25th and 75th percentiles, respectively; the vertical lines indicate the 1.5× the interquartile range; and the colored dots indicate the observed values. (b) The effect size (odds ratio) of the interaction term between treatment and time. Control represents the baseline change in large live tree carbon percentage from 2001 to 2020. Fire, Mech, and Mech+Fire represent the difference between each treatment's change and the baseline change in Control. The vertical line represents no difference. Full model results can be found in Appendix S1: Table S10.

**FIGURE 4:**
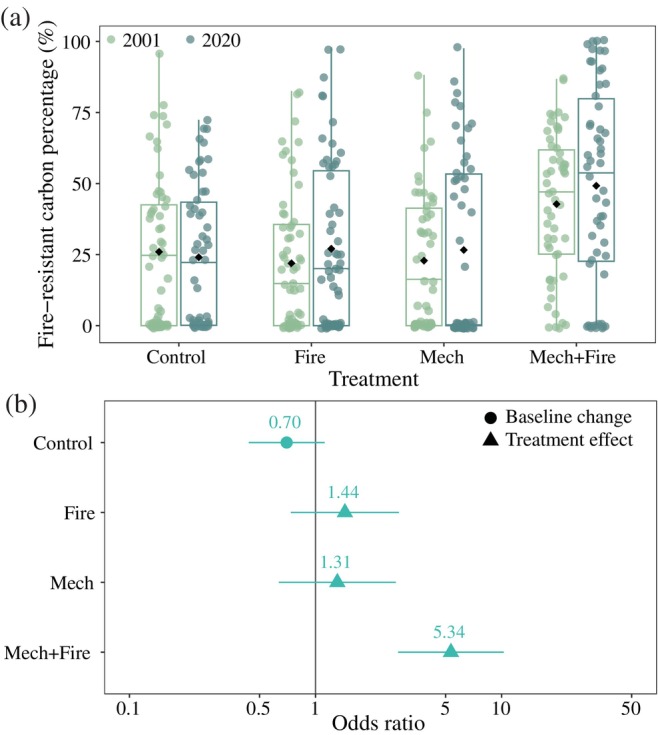
Impact of wildfire mitigation regimes on the species‐specific fire‐resistant carbon percentage (%) at Blodgett Forest Research Station. Species‐specific fire‐resistant carbon refers to carbon stored in fire‐resistant trees (dbh ≥ 11.4 cm), which in this study includes ponderosa pine and sugar pine. (a) Distribution of fire‐resistant carbon percentage in 2001 (before treatment) and 2020 (after treatment). The black dot indicates the plot‐level mean; the midline indicates the median value; the bottom and top of the box indicate the 25th and 75th percentiles, respectively; the vertical lines indicate the 1.5× the interquartile range; and the colored dots indicate the observed values. (b) The effect size (odds ratio) of the interaction term between treatment and time. Control represents the baseline change in fire‐resistant carbon percentage from 2001 to 2020. Fire, Mech, and Mech+Fire represent the difference between each treatment's change and the baseline change in Control. The vertical line represents no difference. Full model results can be found in Appendix S1: Table S11.

On plots where large live tree carbon stocks were greater than 0, treatments that included harvest operations (i.e., Mech and Mech+Fire) experienced significantly greater increases in large live tree carbon percentage (Figure 3b, Appendix S1: Table S10). The percentage of large live tree carbon in Control increased by 10.1 percentage points (2001 = 42.7% ± 3.1%; 2020 = 52.8% ± 3.0%). However, Mech increased by 23.5 percentage points and Mech+Fire increased by 16.5 percentage points over the same interval. In general, there was not only a great deal of plot‐level variation in the large tree fraction but also an excess of zero values in 2020 (Figure 3a, Appendix S1: Table S10). In terms of the percentage of aboveground live tree carbon stored in fire‐resistant species, there was no temporal trend in Control and the only treatment with a significant effect was in Mech+Fire (Figure 4, Appendix S1: Table S11). Between 2001 and 2020, on plots where fire‐resistant carbon was greater than 0, the species‐specific fire‐resistant carbon percentage increased by 17.1 percentage points in Mech+Fire (2001 = 46.2% ± 3.2%; 2020 = 63.3% ± 4.0%).

### Carbon consequences under simulated wildfire

Wildfire emissions were 67%–77% lower in stands that previously experienced fire (Fire or Mech+Fire) compared to stands without active management (Control; Table 3). Although mechanical treatment alone (Mech) almost doubled the emissions relative to the Fire or Mech+Fire units, it still emitted 35% less carbon than Control (Table 3). In terms of tree mortality (P‐mort), Control showed the highest potential tree mortality—4× higher than Fire, 5× higher than Mech, and nearly 7× higher than Mech+Fire. This elevated mortality was reflected in the percentage of dead large‐tree carbon. Control units would lose 28% of their large live tree carbon, compared to less than 5% in all other treatments. Similarly, the percentage of dead fire‐resistant tree carbon is lowest in Mech+Fire (2%), moderate in Fire and Mech (both around 10%), and highest in Control (39%). Active management, especially the combination of mechanical treatment and fire, greatly reduced wildfire‐related mortality in both large trees and fire‐resistant species (Table 3). In absolute terms, the simulated wildfire killed 113 MgC/ha of live tree carbon in Control—about 6× greater loss than Fire and 16× greater loss than Mech+Fire.

**TABLE 3:** Impact of a simulated wildfire event on the treated and untreated stands at Blodgett Forest Research Station.

Treatment	*N*	Wildfire emission (MgC/ha)		% P‐mort		% large tree dead		% fire‐resistant dead		Tree carbon killed by wildfire (MgC/ha)	
		Mean	SE	Mean	SE	Mean	SE	Mean	SE	Mean	SE
Control	53	86	5.0	48	5.6	28	5.7	39	6.4	113	16.3
Fire	55	29	2.5	12	2.4	4	1.7	11	3.6	19	4.2
Mech	46	56	4.8	9	1.0	3	0.4	9	4.0	13	1.3
Mech+Fire	48	20	2.8	7	1.5	2	0.2	2	0.2	7	0.7

*Note*: The wildfire emission was estimated by FOFEM. The %P‐mort was the potential tree mortality measured by the percent of basal area killed and was obtained from the FVS‐FFE potential fire report. The dead large tree carbon and dead fire‐resistant carbon estimates were derived from the FVS‐FFE mortality report (see *Analysis of simulated wildfire*). The %large tree dead was calculated as the carbon in large trees predicted to die during the wildfire divided by the total large live tree carbon before the wildfire. The %fire‐resistant dead was calculated as the carbon in fire‐resistant species predicted to die during the wildfire divided by the total fire‐resistant species carbon before the wildfire. Tree carbon killed by wildfire refers to live tree carbon that would experience mortality due to the simulated wildfire.

Abbreviations: *N*, number of plots; SE, standard error of the mean.

### Comparative carbon cost of treatments

Based on the empirical results (i.e., without wildfire), all treatments incurred a carbon cost compared to Control (Table 4). Among the treatments, Mech had the lowest carbon cost—about 84% the cost of Fire, and 48% the cost of Mech+Fire. The reduced cost associated with Mech was largely due to the longevity of carbon in LLP. Although the total NECB for Mech was 21.7 MgC/ha lower than Fire (Table 2), factoring in the 15.6 MgC/ha stored in LLP reduced its carbon cost (Table 4).

**TABLE 4:** Carbon costs of different wildfire mitigation regimes under empirical non‐wildfire scenario and simulated wildfire scenario at Blodgett Forest Research Station.

Treatments	*N*	Total LLP (MgC/ha)	Undiscounted carbon cost (MgC/ha)		Carbon cost (MgC/ha)	
			Without wildfire	With simulated wildfire	Without wildfire	With simulated wildfire
Control	51	0	…	85.6	0.0	40.6
Fire	40	0	93.9	116.3	74.4	84.9
Mech	39	15.6	93.7	149.8	62.0	88.6
Mech+Fire	44	10.2	172.9	192.1	130.4	139.5

*Note*: Total LLP refers to the sum of carbon stored in LLP after mechanical treatments, with fossil fuel emissions associated with the harvest and transport deducted. LLP is considered as a persistent carbon pool when quantifying the carbon cost. Undiscounted carbon cost is the sum of the net annual differences in carbon storage (includes LLP) between Control and treated units (Control − Treatment) during 2002–2020 (i.e., no discounting applied). Carbon cost represents the sum of the discounted annual differences. A wildfire was simulated in 2020. See Appendix S1: Section S8, Table S5 for details on the calculation of each term. Units in megagrams of carbon per hectare.

Abbreviation: *N*, number of plots.

Based on the immediate impacts of the simulated wildfire, all treatments still incurred a carbon cost compared to Control, although the cost differentials were much smaller (Table 4). Fire replaced Mech as the wildfire mitigation treatment with the least carbon costs. Mech ended up 3.7 MgC/ha higher than that of Fire, partly due to Mech's greater wildfire emissions (Table 3). Still, both Mech and Fire were about 40% less costly than Mech+Fire. These patterns in carbon costs across treatments were consistent without discounting (i.e., undiscounted carbon cost, Table 4), but the magnitude of the differences was much less.

The longer term consequences of the simulated wildfire on carbon cost were not modeled. However, wildfire dramatically changed where carbon was stored in Control. It shifted 113 MgC/ha of aboveground, live tree carbon to aboveground, dead tree carbon (Table 3). This transfer from live‐to‐dead tree carbon was an order of magnitude greater than any of the mitigation treatments.

## DISCUSSION

### Carbon dynamics of wildfire mitigation regimes

The sustained, 19‐year regime of wildfire mitigation treatments unequivocally reduced carbon stocks at Blodgett Forest compared to the controls (Figure 1). This reduction in carbon storage is a consistently documented and widely recognized consequence of wildfire mitigation efforts in the frequent‐fire forests of the American West (Kalies & Kent, 2016; Wright et al., 2021). However, differences among the treatments point to key differences in carbon dynamics. For example, even though Fire emitted 2.1× more carbon than Mech (Table 2), the net ecosystem carbon balance (NECB) in Fire was positive (19.6 MgC/ha) while Mech lost carbon (NECB = −2.1 MgC/ha). The driver of this discrepancy was NEP; the annual NEP in Fire was 2.7× greater than in Mech (Table 2). Although emissions were higher in Fire, most were produced by the combustion of litter, duff, and fine woody debris. In contrast, carbon losses in Mech were mainly due to the reduction in the live tree pool (Appendix S1: Table S6). Thus, Fire suffered a much lower reduction in photosynthetic capacity compared to Mech.

Wildfire mitigation treatments are expected to increase NEP relative to controls (Campbell et al., 2012; Niu et al., 2024), but results from this study did not follow expectations. The basis for the expected productivity bump is that moderate severity disturbances related to treatments increase resource availability and/or resource use efficiency (Curtis & Gough, 2018). For example, at a comparable site in the southern Sierra Nevada (Wiechmann et al., 2015), the burned units averaged 5.3 MgC/ha/year during the 9 years after treatment (2002–2011), while the control units had effectively 0 NEP (−0.02 MgC/ha/year) during the same interval. In contrast, Control at Blodgett maintained the highest annual average NEP over the last 20 years (Table 2) despite maintaining high levels of inter‐tree competition throughout the study (Table 5, Appendix S1: Table S2). Among treatments, Fire generated much higher NEP than either mechanical treatment (Table 2). The fact that Fire maintained higher live tree carbon (Appendix S1: Table S6) certainly contributed to its greater productivity. Another important factor was the addition of nutrients associated with repeated entries. Given its consumption of surface fuels, prescribed fire can mobilize major soil nutrients (Boerner et al., 2009; Certini, 2005). For example, Moghaddas and Stephens (2007) found that the 1st entry fire increased available mineral soil nitrogen (NO_3_^−^ and NH_4_^+^) by an order of magnitude larger than the 1st Mech entry. The greater nutrient availability at Blodgett may have supported the disproportionate NEP response in Fire as opposed to Mech.

**TABLE 5:** A summary of the relative benefits and costs of wildfire mitigation strategies at Blodgett Forest Research Station.

Treatments	Fire hazard (% P‐torch)		Forest health (% SDI_max_)		Economic cost (US$/ha)		Carbon cost (MgC/ha)				Stable carbon (MgC/ha)	
							Without wildfire		With simulated wildfire			
	Mean	Rank	Mean	Rank	Mean	Rank	Mean	Rank	Mean	Rank	Mean	Rank
Control	65	4	75	4	0	3	0	1	40.6	1	43.8	4
Fire	25	3	51	3	2536	4	74.4	3	84.9	2	52.5	3
Mech	19	2	44	2	−5023	1	62.0	2	88.6	3	60.3	2
Mech+Fire	2	1	34	1	−64	2	130.4	4	139.5	4	69.9	1

*Note*: Forest health and economic cost were obtained from Stephens et al. (2024). Like carbon cost, the economic cost was discounted to 2001 based on the US inflation rate. Fire hazard and forest health report values for 2020. Stable carbon is the aboveground, live tree carbon stored in large, fire‐resistant individuals that are expected to survive after a wildfire (Appendix S1: Table S14). Note that the means reported for fire hazard were averaged across all the plots, including plots with zero.

Early indications from the same study at Blodgett Forest (Dore et al., 2016) and a comparable study in the southern Sierra Nevada (Hurteau & North, 2010) concluded that fuel reduction treatments may have a net negative impact on carbon storage in the short term. However, results presented in this work demonstrate that wildfire mitigation efforts can sequester carbon. After two decades of treatments, the NECB for Fire was positive (Table 2). If LLP from Mech are counted as a stored carbon pool (Table 4), Mech also proved to be a small carbon sink (−2.1 MgC/ha in NECB + 15.6 MgC/ha in LLP = 13.5 MgC/ha). Among the treatments, only Mech+Fire was a net carbon source (−70.7 MgC/ha in NECB + 10.7 MgC/ha in LLP = −60 MgC/ha).

### Post‐fire recovery

One concern with prescribed fires as a wildfire mitigation treatment is the potential for rapid recovery of surface fuels, as fire‐killed trees fall and scorched crowns resupply litter and fine woody debris. The mass of the fuel bed represents the balance between inputs (leaves and branches) and outputs (decomposition, Keane, 2016). Scorched leaves and delayed mortality after prescribed fires, especially initial‐entry fires, can result in pulsed additions to the fuel bed (Kennedy et al., 2021). Moreover, any increase in productivity following fire (Figure 2a–c) would only add to the surface fuel load. Based on a longitudinal study following low‐severity wildfires in dry conifer forests of California, fine fuel recovery peaked about 6 years after the fires (Eskelson & Monleon, 2018). At Blodgett, the rate of surface fuel recovery was fast enough that by 2009 (7 years post‐treatment), surface fuel loads in Fire were statistically indistinguishable from those in Control (Foster et al., 2020). While the absolute rates of surface fuel recovery continued to increase after the 2nd and 3rd entries, the rates were slower in Fire than in Control (Figure 2d,e).

The temporal trends in the accumulation and recovery rate of the aboveground live vegetation suggest that NEP has shifted over the course of the study (Figure 2). As Dore et al. (2016) noted, the carbon accumulation rate in Control significantly outpaced the carbon recovery rate in Fire after the 1st entry (Figure 2a). After the 2nd entry, carbon recovery in Fire was much faster, matching the rate in Control (Foster et al., 2020, Figure 2b). However, by the 3rd entry, the pattern had reversed, and the carbon accumulation rate in Fire outpaced Control (Figure 2c). The reversal was due in part to successively increasing recovery rates of aboveground live vegetation carbon following each prescribed fire. But the accumulation rate of live vegetation carbon in Control also declined steeply over the last three intervals. These results suggest that the increased productivity spurred by a frequent‐fire regime does not cause an equally rapid rise in surface fuels. However, these carbon accumulation and recovery trends must be interpreted cautiously.

A potential driver of this divergence may be treatment‐level differences in the drought response. California experienced an extreme drought between 2012 and 2015 that spurred extensive tree mortality in the central and southern Sierra Nevada (Fettig et al., 2019). A major contributor to the mortality was a drought‐induced outbreak of bark beetles that caused widespread death in 2015 and 2016 (Fettig et al., 2019). A long‐term study in the southern Sierra Nevada found increased live tree carbon during 2002–2011, but decreased live tree carbon during 2012–2017 regardless of the treatments (Goodwin et al., 2020). The drought was considerably less severe in the north‐central Sierra Nevada, with relatively little bark beetle activity at Blodgett Forest and only minor mortality increases (Axelson et al., 2019). A drought of a similar severity to that experienced in the southern Sierra Nevada would only accentuate the impact of tree competition on NEP. At the start of the study, the aboveground live tree carbon in Control was in the 87th percentile for all California mixed conifer forests (Forest Inventory and Analysis, 2024). By 2020, it was at the 96th percentile with a mean relative SDI in the range of “imminent mortality” (North et al., 2022; Stephens et al., 2024). Indeed, the annual tree mortality in Control steadily increased with each interval: 0.9%/year in the 1st interval to 3.2%/year in the 3rd. In Fire, tree mortality steadily declined from 4.7%/year to 2.7%/year (1st to 3rd interval, Appendix S1: Table S12). Given the link between mortality and growth (Cailleret et al., 2017), reduced tree growth post‐drought could explain the slowing rate of NEP in Control. However, a direct comparison of drought impacts on tree growth is confounded by the coincident timing of the drought and the 3rd entry burn. The 2016–2020 carbon dynamics in Fire are likely influenced by both drought and prescribed fire.

It appears that the rate of carbon accumulation in Control is slowing as it approaches culmination (Smith & Long, 2001). This pattern of NEP with time since disturbance follows standard expectations for a temperate conifer forest (Gower, 2003). In contrast, a regime of repeated prescribed fires seems to have maintained carbon storage at rates high enough to offset fire emissions (Stephens et al., 2024).

### Wildfire‐resistant carbon

Managing forests to reduce the vulnerability of carbon to wildfire or other disturbances is an important policy objective (Fargione et al., 2018). Soil and live trees are the two dominant carbon pools of the global forest carbon stock (Mo et al., 2023). This pattern held for Blodgett Forest. In 2020, the aboveground live tree carbon in Control averaged 245 ± 14.7 MgC/ha (Appendix S1: Table S6). In 2018, the mineral soil carbon to 90 cm depth in a comparable stand at Blodgett was estimated as 186 ± 30.1 MgC/ha (Soong et al., 2021). All mineral soil carbon (Jandl et al., 2007) and the fraction of carbon in large trees of fire‐resistant species (Wiechmann et al., 2015) represent carbon that is relatively stable in a mixed‐severity surface fire regime.

Recent reviews concluded that thinning (Mäkipää et al., 2023) and prescribed fire (Alcañiz et al., 2018) have minimal impacts on mineral soil carbon in most forest ecosystems. A meta‐analysis of disturbance impacts on forest soil organic matter in the Pacific Northwest reached similar conclusions (Nave et al., 2022), but few studies tracked the impact of repeated entries of fire on soil properties (Fontúrbel et al., 2021). The initial treatments in this study had no immediate impact on carbon in the top 15 cm of the mineral soil (Moghaddas & Stephens, 2007), and no difference was reported for mineral soil carbon (0‐ to 15‐cm layer) between Control and Fire after the 2nd entry (Dore et al., 2016). However, between 2001 and 2020, there was a significant increase in mineral soil carbon in the top 15 cm of Control (58.7–71.2 MgC/ha), while no change was reported for the treatments (Appendix S1: Table S6). The increase in SOC was due to a consistent increase in the carbon content in Control from 5.07 %C in 2001 to 5.57 %C in 2020, accompanied by large increases in litter and duff carbon (27.7–46.6 MgC/ha, Appendix S1: Table S6). The relative difference between controls and treatments aligns with a comparable study in the southern Sierra Nevada where prescribed fires had been applied three times every 10–15 years (Pellegrini et al., 2021). In 2017, soil carbon between 0 and 15 cm depth was 41.8 MgC/ha in the prescribed fire regime and 53.7 MgC/ha in a nearby unburned forest. These results suggest that a restored disturbance regime may reduce carbon storage in the upper mineral soil relative to forest stands subject to prolonged fire exclusion. However, in both cases, the absolute changes in mineral soil carbon were modest.

While the primary goal of the wildfire mitigation treatments was to alter fire behavior by reducing tree density and surface fuel loads, they also shifted tree size distribution (Stephens et al., 2024) and species composition (Figure 4a). Together, these shifts implied that the treatments support robust per capita increases in tree carbon, with the gains concentrated in the large trees. Although the treatments led to only small increases in the absolute density of large trees (dbh > 76.2 cm, Stephens et al., 2024), the relative large tree density in the treated units increased greatly. Large trees accounted for 9.7% ± 1.1% of the number of live trees in Control in 2020 but 22.7% ± 3.1% in Fire, 28.2% ± 2.8% in Mech, and 35.4% ± 4.7% in Mech+Fire. By 2020, on average, the large trees stored 48% or more of the aboveground live tree carbon pool in the treatments (Figure 3a). At the same time, tree competition declined (Stephens et al., 2024). As reported by Stephenson et al. (2014), larger trees sustainably accumulate carbon at a faster rate than smaller trees. The impact on growth is likely to be accelerated, given the observed decreases in competition (Das, 2012). Finally, among the conifer species in the study, the two fire‐resistant species—ponderosa pine and sugar pine—also have the highest growth efficiency (Gersonde & O'Hara, 2005). In summary, the observed trend in live vegetation accumulation in Fire (Figure 2a–c) fits our current understanding of vegetation dynamics in these stands. Research to quantify the underlying drivers of the individual tree growth response to treatments is ongoing.

### Avoided emissions

The impact of the simulated wildfire on carbon dynamics at Blodgett Forest (Table 3) matched well‐documented expectations: all treatments substantially reduced emissions during wildfire compared to Control (Restaino & Peterson, 2013). Among treatments, the results also highlighted the value of prescribed fire in limiting emissions. Despite similar reductions in fire severity as measured by the probability of tree mortality (%P‐mort, Table 3), both fire treatments (i.e., Fire and Mech+Fire) produced less than half the emissions of Mech. Specifically, the ratio of emissions to %P‐mort ranged from 2.4 for Fire and 2.8 for Mech+Fire to 6.2 for Mech (Table 3). A source of the added emission for Mech was the much higher pre‐fire litter and duff carbon. In 2020, prior to the simulated wildfire, Mech stored 2.6× more carbon in litter and duff than Fire and 2.8× more than Mech+Fire (Appendix S1: Table S6). During the simulated wildfire, the combustion of these surface and ground fuels in Mech added 19.7 MgC/ha more emissions than Fire and 21.1 MgC/ha more than Mech+Fire. Without recent fire, litter and duff are typically the largest carbon pools consumed and the major source of emissions during a wildfire (e.g., Campbell et al., 2007). Duff, given its propensity to combust under smoldering conditions, can contribute disproportionately to the smoke emissions during fire (e.g., Zhao et al., 2019). Thus, despite only minor differences in fire hazard reduction among treatments, Mech carried a notably higher potential emission burden.

### Carbon costs

The carbon cost comparison (Table 4) extended the analysis beyond the stock‐change framework used to estimate the forest carbon balance (Table 2). Specifically, the calculations accounted for the emissions produced by the three prescribed fires and the carbon stored in wood products. In addition, the discounting standardized the opportunity costs of atmospheric emissions to a 2001 baseline. Under this accounting framework, avoided emissions from a simulated wildfire after 19 years of restoration did not offset the carbon losses from mitigation treatments (Table 4). This result aligns with conclusions from a simulation model (Campbell et al., 2012) and a sensitivity analysis for a similar fire‐prone, conifer forest in the Pacific Northwest (Campbell & Ager, 2013). Due to the infrequency of wildfire–treatment interactions, the mitigation efforts did not increase carbon storage relative to the control.

Despite the value of these data‐driven results, their inferential breadth is limited in space (stand‐scale) and time (last 20 years). The carbon benefit depends on disturbance regime dynamics. On the one hand, there is the current regime of active fire suppression that leads to infrequent but high‐severity disturbances (Steel et al., 2015). On the other hand, there is a prospective future regime where wildfire mitigation treatments impose frequent but low‐severity disturbances (Campbell et al., 2012). Given the need to evaluate counterfactual scenarios (i.e., estimating the probability and severity of wildfire) and potential disturbance regimes (i.e., repeated wildfire mitigation treatments), calculating the carbon benefit of avoided emissions, including those associated with wildfire‐driven forest conversions (Coop et al., 2020), relies on outputs from forest growth and dynamics models, fire behavior and effects models, projections of future climate, and predictions of burn probability (e.g., Foster et al., 2020; Liang et al., 2018; Loudermilk et al., 2017). Considering the extensive empirical record and the well‐documented modeling frameworks, a promising way forward to improve our understanding of fire‐carbon dynamics involves tighter integration between field measurements and ecosystem models (i.e., data‐model integration, Niu et al., 2014).

### Management implications

Management of frequent‐fire forests in the American West poses a classic challenge—how to balance competing objectives (Bradford & D'Amato, 2012). And yet, given the extent of anthropogenic impacts on forest dynamics, this “balancing act” is growing more complex and uncertain (Seidl et al., 2017). After addressing the legal and operational constraints to treatment options (sensu Davis et al., 2024), a key trade‐off is the carbon costs of a management regime designed to reduce wildfire hazard (e.g., Hurteau et al., 2011).

The 20‐year experiment at Blodgett Forest provides a unique opportunity to quantify the costs and benefits of different pathways. To this end, results from this study and from Stephens et al. (2024) were summarized to evaluate the management trade‐offs (Table 5). The dimensions of the decision space are best illustrated by comparing the extremes. The most intensive treatment, Mech+Fire, delivered the largest wildfire hazard reduction, the greatest improvement in forest health, and the highest carbon stability, while accumulating the highest carbon costs. The least intensive treatment, Control, incurred the lowest carbon costs but was exposed to the greatest wildfire hazard, experienced the most competitive stress, and maintained the smallest stable carbon (Table 5). This contrast reveals a key trade‐off between carbon cost and carbon stability. Assuming that the most wildfire‐resistant subset of trees is large pines (Table 1), Control included only 44 MgC/ha in this stable pool (Table 5, Appendix S1: Table S14)—62%–83% less than the treatments. In economic terms, both Control and Mech+Fire were inexpensive to implement. Moreover, these rankings were consistent under both prevailing and simulated conditions.

The Fire and Mech pathways fell between these two extremes in terms of wildfire hazard reduction, forest health, stable carbon storage, and carbon costs. In relative terms, the simulated wildfire had only a modest impact. Mech had 25% lower carbon costs than Fire without a wildfire and 4% higher costs with a wildfire (Table 5). Finances were the big difference between these treatments. Mech generated revenue; Fire incurred expenses (Table 5). The net differential, adjusted for inflation, was $7559/ha.

There were also benefits and costs not quantified here. Among the treatments, prescribed fire tends to provide unique ecological benefits (Ryan et al., 2013). For example, the Fire units at Blodgett Forest supported fewer introduced species than Mech or Mech+Fire (Dudney et al., 2021). Also, treatments that only rely on mechanical thinning can lead to comparatively higher surface fuel loads (Vaillant et al., 2009). As noted above, Mech produced more smoke than either Fire or Mech+Fire for a comparable fire hazard reduction (Table 3). Post‐wildfire consequences were also not captured, including the potential for tree regeneration failure (e.g., Davis et al., 2019, Nagelson et al., 2024), and severe reburning in short succession (e.g., Coppoletta et al., 2016). With regard to the specific results presented here, the simulated wildfire created more precarious near‐term conditions in Control. After simulated wildfire, Control had an order of magnitude more dead tree carbon (113 MgC/ha, Table 3). Thus, going forward, Control would likely encounter rising heavy fuel loads (i.e., 1000‐h) from degrading, fire‐killed snags (Grayson et al., 2019) while also storing less stable carbon.

Insights from this retrospective synthesis do not account for the expected impacts of a warming and drying climate. The changing climate in the Western United States will likely generate more frequent and more severe wildfires, which will inevitably shift the cost–benefit balance of mitigation treatments (Wasserman & Mueller, 2023). For example, Loudermilk et al. (2017) modeled the carbon balance of mitigation treatments comparable to Mech and Mech+Fire conducted across a Sierran mixed conifer landscape. Their landscape simulations were run under two different climate change scenarios with results compared to the climate baseline (i.e., observed climate from 1960 to 1990). For the first three decades (2020–2050), their results qualitatively matched our results: the carbon removals due to treatments exceeded the avoided wildfire emissions. However, after 2050, the carbon storage curve “bends” to favor the treatment scenario. In a companion study, Krofcheck et al. (2017) used the same modeling framework and found the increased frequency of extreme fire weather was the main driver of carbon balance among mitigation treatments, with Mech+Fire showing the lowest carbon costs under such conditions. Given the slow dynamics of forests relative to the projected climate changes, all mitigation options must be evaluated for both near‐term and longer‐term efficacy.

An essential insight from this long‐term experiment is the need to sustain a management strategy that mimics the natural disturbance regime, namely one with frequent, low‐intensity biomass removal. Affordability is a major constraint. In the non‐wildfire scenario, Mech+Fire's trade‐off between wildfire hazard reduction and carbon costs is asymmetrical. Mech+Fire reduced wildfire hazard by an order of magnitude, but also doubled the carbon costs (Table 5). In the wildfire scenario, it had the lowest wildfire emission, but its carbon cost remained 60% higher than Mech or Fire alone. As the most effective wildfire mitigation strategy (Davis et al., 2024), Mech+Fire might be the preferred option in situations where reducing fire hazard is the top priority, for example, to protect communities, firefighters, or highly valued natural resources.

There are opportunities to reduce the carbon costs of Mech and Mech+Fire treatments. Of the carbon harvested in Mech, on average 37% was unused residue left in the field or piled and burned at the site (i.e., tops, branches, and leaves), 14% was low‐value wood such as bark and small‐diameter fuelwood, and 16% was lost during processing in the mill (Appendix S1: Table S15). In the end, only 33% of harvested logs were made into LLP. California‐wide statistics presented a similar ratio of unused residues (32%) and low‐value wood (20%, Christensen et al., 2021; Khatri et al., 2023). Developing products that use these harvest residues and low‐value wood could lead to greater returns in carbon, but how much such innovative use reduces carbon costs depends on the product. Residue used for biopower has no long‐term storage value but could offset emissions from other power sources. In contrast, residue incorporated into engineered wood products (e.g., oriented strand board, cross‐laminated timber) has higher substitution benefits or higher carbon storage density (Cabiyo et al., 2021). The economic return is also high; for instance, Cabiyo et al. (2021) valued harvest residue delivered for low‐carbon fuel and oriented strand board production at more than $100 per oven‐dried megagram.

It is increasingly important to account for the health costs of smoke exposure in fire management decisions and future trade‐off analysis. In recognition of the growing evidence on the dangers of particulate pollution, the U.S. Environmental Protection Agency recently lowered the annual PM2.5 standard (Environmental Protection Agency, 2023). Though both wildfires and prescribed fires produce smoke, their health impacts differ based on differences in wind and weather, fuel composition, combustion completeness, and the presence of anthropogenic materials (Aguilera et al., 2021; Jaffe et al., 2020). The planned nature of prescribed fires provides opportunities to reduce smoke exposure by individuals and communities. Implementing prescribed fire under conditions that minimize smoke exposure can help minimize health impacts (Kiely et al., 2024).

Long‐term field experiments provide valuable insights into ecosystem processes and temporal dynamics. When the experimental treatments reflect real‐world practices, they also inform resource management and policy (Knapp et al., 2012). However, one of the “perils” of long‐term experiments is that they are often conducted at a single site and may not be representative of the area of interest (Fahey et al., 2015). Blodgett Forest is a productive site in the California mixed conifer forest type. Its deep (150 cm), well‐drained, nutrient‐rich soil supports rapid tree growth (Busse et al., 2021). Olson and Helms (1996) estimated that its favorable conditions reflect only 7% of the vast (>31,000 km^2^) mixed conifer forest. Its flat terrain and extensive road network also reduce the operational burden of treatments. The cost of wildfire mitigation treatments, both in terms of carbon and dollars, is likely to be greater in less productive, steeper, and infrastructure‐limited forests.

Another peril of long‐term field research is that ambient conditions cannot be controlled. The longevity of the study increases the chance that infrequent events (e.g., droughts, wildfires, and invasive species) will intersect the planned experiments (Knapp et al., 2012). While these disturbances may provide opportunities for new ecological insights, they complicate the interpretation of treatment effects. In this case, the 3rd Fire and the 2nd Mech and Mech+Fire treatments occurred at the end of an extended hot drought (2012–2015). Disentangling drought impacts from the treatments on the carbon balance would require a more temporally explicit analysis—a challenge left for future research.

## CONCLUSION

Restoring key elements of a disturbance regime—frequent, low‐intensity biomass removal—significantly reduced wildfire hazard (Stephens et al., 2024). This regime effectively stored more carbon in live trees than estimated for historical Sierra Nevada forest conditions (Bernal et al., 2022). Compared to the maximum estimate of aboveground live tree carbon (38 MgC/ha, Supplementary tab. 2 in Bernal et al., 2022), the treatments stored 3.5× (Mech+Fire), 4.5× (Mech), and 4.9× (Fire) more carbon (Appendix S1: Table S6) than similar forests prior to Euro‐American settlement. These findings highlight the value of sustained wildfire mitigation in confronting the challenges posed by a warming world. All three pathways reduced fire risk while limiting contributions to greenhouse gas emissions.

## AUTHOR CONTRIBUTIONS

**Yihong Zhu:** Conceptualization; methodology; formal analysis; data curation; validation; writing – original draft; review and editing; visualization. **Daniel E. Foster:** Data curation; methodology; investigation; writing – review and editing. **Brandon M. Collins, Scott L. Stephens, and Robert A. York:** Project administration; funding acquisition; conceptualization; writing – review and editing. **Emily E. Y. Moghaddas:** Investigation; writing – review and editing. **Ariel T. Roughton and John E. Sanders:** Project administration; investigation; data curation. **John J. Battles:** Conceptualization; methodology; formal analysis; validation; resources; writing – review and editing; supervision; project administration; funding acquisition.

## CONFLICT OF INTEREST STATEMENT

The authors declare no conflicts of interest.

## Supporting information


Appendix S1.

## Data Availability

Raw data of the Fire and Fire Surrogate Study in Blodgett Forest Research Station (Stephens et al., 2025) are available in the Environmental Data Initiative Data Portal at https://doi.org/10.6073/pasta/06a3a2556f5289b89b9f8683becfe08e. Processed data and analytical code (Zhu et al., 2025) are available on Zenodo at https://doi.org/10.5281/zenodo.16756682.
