# Supplementary material for: Carbon costs of different pathways for reducing fire hazard in the Sierra Nevada
Source: Ecol Appl. 2025 Nov 2;35(7):e70111. doi: 10.1002/eap.70111 (PMC12580073; doi:10.1002/eap.70111)
Supplement: Supplementary file 1 — Appendix S1. [file EAP-35-e70111-s001.pdf]

Journal: Ecological Applications

Title: Carbon costs of different pathways for reducing fire hazard in the Sierra Nevada

Authors: Yihong Zhu, Daniel E. Foster, Brandon M. Collins, Scott L. Stephens, Robert A. York, Ariel T. Roughton, Emily E.Y. Moghaddas, John E. Sanders, John J. Battles

Appendix S1: Supplementary methods and results

Supplementary methods

Section S1. Site description

This experiment was performed at the Blodgett Forest Research Station (Blodgett Forest), approximately 20 km east of Georgetown, California (38°54’45’’ N, 120°39’27’’W). The main property of Blodgett Forest is 1,780 ha in area and spans an elevation gradient between 1,100 and 1,410 m above sea level. Soils at Blodgett Forest are well developed, well drained Haploxeralfs (Alfisols) derived from either andesitic mudflow or granitic/granodiorite parent materials (Moghaddas and Stephens 2007). The terrain is moderate with average slope less than 30%. The climate is Mediterranean with a summer drought period that extends into the fall. Winter and spring receive the majority of precipitation. From 1994 to 2020, the mean precipitation during winter and spring was 145 cm/year (York et al. 2021). Mean daily temperature was 6.2 °C, mean daily low temperature was 2.6 °C and mean daily high temperature was 10.9 °C (York et al. 2021). Summer months are mild with mean daily August temperatures between 10 and 29°C, with infrequent summer precipitation from thunderstorms (Stephens et al. 2023).

Blodgett Forest supports a productive California mixed conifer forest, a forest type typical for the mid-slope region on the west side of the Sierra Nevada. Species dominance is shared by six tree species (Table S1): white fir (*Abies concolor*), incense-cedar (*Calocedrus decurrens*), Douglas-fir (*Pseudotsuga menziesii*), ponderosa pine (*Pinus ponderosa*), sugar pine (*Pinus lambertiana*), and California black oak (*Quercus kelloggii*). At the start of the study (2001), tree basal area ranged from 48.5 to 54.7 m^2/ha (Table S2). Forested areas at Blodgett Forest have been repeatedly harvested and subjected to fire exclusion and suppression for the last 120 years — a management history shared by many forests in California and elsewhere in the

western US (Graham, McCaffrey, and Jain 2004). Fire was common in the mixed conifer forests of Blodgett Forest before the removal of Indigenous stewardship. Between 1750 and 1900, median composite fire intervals at the 9 – 15 ha spatial scale were 4.7 years with a fire interval range of 4 – 28 years (Stephens and Collins 2004).

Section S2. Experimental design

At Blodgett Forest, 12 similar experimental units were selected for inclusion in the national Fire and Fire Surrogate Study (FFS). The units ranged in size from 14 to 29 ha for a total of 225 ha. Three replicates of four treatments were randomly assigned to units. Pre-treatment measurements confirmed that units were comparable in terms of forest composition and structure (Table S1, Table S2). The four treatments – control, prescribed fire, mechanical treatments, and mechanical treatments followed by prescribed fire – were initially installed in late 2001 and 2002 (Stephens and Moghaddas 2005). Treatments were designed to reduce fire severity using management practices common to the northern Sierra Nevada (Agee and Skinner 2005; Schwilk et al. 2009). The primary objective was to modify the stand structure such that 80% of the dominant and co-dominant trees in the post-treatment stand would survive a wildfire model under 80th percentile weather conditions (McIver et al. 2012; Skinner and Stephens In press). The secondary objective was to create a stand structure that maintained or restored several forest attributes and processes including, but not limited to, snag and coarse woody debris recruitment, floral and faunal species diversity, and seedling establishment. For a detailed summary of the FFS, see Stephens (2021).

The experiment used a Before-After-Control-Impact design (BACI, Stewart-Oaten and Bence 2001). This design explicitly controls for pre-impact differences among the units. As documented by Christie et al. (2019), this design provides statistical power and robustness on par

with randomized controlled trials. The specifics of each treatment are outlined below; more details are available in Stephens and Moghaddas (2005) and Stephens et al. (2009).

Control units (Control) received no treatment during the study period. Wildfires were successfully excluded for the interval reported in this study (2001-2020). Note that while the 2022 Mosquito Fire burned through one of the three control units, our results predate this event.

Fire-only units (Fire) were burned with no mechanical pretreatment using strip head fires three times. The 1st entry was in Fall 2002; the 2nd entry was in Fall 2009; and the 3rd entry was Fall 2017. Burn plans prescribed the following weather parameters: air temperature between 0 – 10°C; relative humidity >35%, and wind speed < 5 km/h. The desired 10-h fuel stick moisture content was 7% – 10%.

Mechanical-only treatment units (Mech) experienced two treatments, both of which include a two-stage prescription. The first one was in 2001. Units were first crown thinned and then thinned from below to remove ladder fuels. While the crown thinning removed trees in the upper canopy, generally the largest trees were retained. Thinning guidelines emphasized retaining large trees in an even mix of conifer species in the after-treatment forest (Stephens and Moghaddas 2005). Individual trees were felled, bucked, and limbed using a chainsaw and boles then yarded to landings with ground-based skidders. The sawlogs were transported to a sawmill; tops and limbs were masticated and left to decompose. Generally, residual trees were well spaced with little overlap of live crowns of the dominant and co-dominant trees. The thinning step retained 28–34 m^2/ha of basal with larger-diameter trees contributing most of the remaining basal area.

Following the harvest, approximately 90% of understory conifers and hardwoods up to 25 cm diameter at breast height (breast height = 1.37 m, DBH) were masticated in place using an

excavator-mounted rotary masticator. A second Mech-only treatment was done with understory mastication in 2017 followed by a second thinning from below in 2019. This second thinning operation incorporated mechanized harvesting and yarded whole trees to landings, thus leaving less of the harvest-related fuels ("activity fuels") in the units. In both operations, treatments were specifically designed to reduce tree density while also reducing surface fuels by limiting fuel input over time (i.e., removing trees that would have died from competition). And the mastication treatments both removed ladder fuels and sped up their decomposition.

Mechanical+Fire units (Mech+Fire) underwent the same initial treatment as Mech units in 2001, but following mastication they were broadcast burned using a backing fire in the fall of 2002. The fire consumed large amounts of activity fuel that came from mastication of medium sized trees and harvesting slash. In 2017, shrubs and small trees that developed following 2002 were masticated a second time (similar to Mech), but larger trees were not thinned given their low levels of stocking. The units were then burned with strip head fires in 2018. The fire consumed the activity fuel that came from mastication of shrubs and small trees. After this second fire, salvage harvesting of clumps of fire-killed trees occurred in 2019 in the Mech+Fire units. The two harvest operations in Mech+Fire followed the same procedures of the two used in the Mech.

Wildfire simulations were applied to each unit with inputs based on the 2020 forest and fuel conditions. Model implementation replicated the procedures used in Stephens et al. (2024). Fuel model inputs and the target indices were derived using a weighted average from the two "best" fuel model assignments given the measured fuel conditions. Weather for each simulation was under "severe" conditions: wind speed = 32 km h^{-1}, air temperature = 21°C, 1-h fuel

moisture = 3%, 10-h fuel moisture = 4%, 100-h fuel moisture = 5%, 1000-h fuel moisture = 10%, duff fuel moisture = 15%, and live fuel moisture (woody and herb) = 70%.

Section S3. Carbon terminology

Given the plethora of terms used to describe carbon dynamics across disciplines, the definitions for terms used in this study are defined below.

S3.1 Total measured carbon

Total measured carbon is the sum of all carbon pools measured in the field, namely aboveground live tree, standing dead tree, understory vegetation, coarse woody debris (>1000-h fuels), fine woody debris (1-h, 10-h, and 100-h fuels), litter and duff, and mineral soil (0 – 15cm). This term is used to measure the impact of the treatments on total forest carbon stock.

S3.2 Total net ecosystem carbon balance

Total net ecosystem carbon balance (NECB) is the change in total measured carbon between 2001 and 2020. NECB is calculated using a stock change approach based on the measured pools. Note that two major pools, belowground tree carbon (i.e., coarse roots) and mineral soil carbon deeper than 15 cm, are not included in this estimate.

S3.3 Net ecosystem productivity

Total net ecosystem productivity (NEP) is based on the change in measured carbon pools between 2001 and 2020 (NECB) with the estimated emissions from Fire treatments and the wood removals from Mech treatments over the 2001-2020 interval included (NEP = NECB - Fire emission - Wood removals). Annual NEP is the total NEP divided by the measurement interval of 19 years. As noted above (Section S3.2), NEP is calculated using a stock change approach and does not include belowground tree carbon and mineral soil carbon deeper than 15 cm.

S3.4 Live vegetation recovery/accumulation

The annual change in the aboveground live vegetation carbon pool following prescribed fire is defined as the vegetation recovery rate. This rate was compared to the trajectory of carbon accumulation in the Control units. Specifically, the rate of carbon recovery (Fire) or accumulation (Control) in the aboveground live vegetation (i.e., sum of tree, shrub, and herb) was expressed as the annual average change in carbon density (MgC/ha/yr) during the three intervals following the application of prescribed fire. Each recovery period began immediately after a fire and continued until the next fire (1st recovery period: 2003 – 2009, 2nd: 2010 – 2016, 3rd: 2017 – 2020).

S3.5 Surface fuel recovery/accumulation

The annual change in the surface fuel carbon pool following prescribed fire is defined as the fuel recovery rate. This rate was compared to the trajectory of the fuel accumulation in the Control units. Specifically, the rate of carbon recovery (Fire) or accumulation (Control) in the surface fuel (i.e., sum of litter, 1-h, 10-h, and 100-h fuels) was expressed as the annual average change in carbon density (MgC/ha/yr) during the three intervals following the application of prescribed fire. These recovery periods are consistent with that of live vegetation recovery (1st recovery period: 2003 – 2009, 2nd: 2010 – 2016, 3rd: 2017 – 2020).

S3.6 Wildfire-resistant carbon

The vulnerability of trees to wildfire loss varies by size and species (Cansler et al. 2020; Stevens et al. 2020). The difference in these vulnerabilities was used to define subsets of the aboveground live tree pool that were more resistant to wildfire loss. One wildfire-resistant pool included large trees (DBH ≥76.2 cm) of all species (large tree pool); the other included all

ponderosa and sugar pine trees (DBH $\geq$ 11.4 cm) in the live tree carbon pool (Van Mantgem and Schwartz 2003; Rodman et al. 2020).

S3.7 Fire emissions

Fire emissions are defined as the atmospheric carbon emissions (e.g: CO_2, CO, and CH_4) from each prescribed fire.

S3.8 Long-lived wood product

Long-lived wood products (LLP) refer to the carbon from harvested trees that is stored in long-lived wood products (e.g., dimensional lumber, decking, and furniture). To account for the fossil fuel emissions associated with the harvest and transport, 5% of carbon stored in LLP was deducted from this pool when quantifying the carbon cost (Buchholz et al. 2021).

S3.9. Total wood removals

Total wood removals refer to the carbon removed from the forest during harvest operations. It includes sawlogs milled to LLP, wood harvests used for fuel, and the offsite (i.e., at the landing) disposal of harvest debris.

S3.10 Carbon cost

To evaluate the carbon costs of management, Peng et al. (2023) emphasized taking forest regrowth after management operations into account by tracking annual fluxes (emission or sequestration) compared with Control and discounting its value by time. The carbon cost of treatments represented the magnitude of carbon storage (MgC/ha) in each year that was not realized due to the treatments. In other words, the cost of per-unit emissions is equivalent to the absolute value of per-unit mitigations. However, given the assumption that mitigation will become less expensive in the future (Daniel, Litterman, and Wagner 2019; Wilberforce et al. 2021), the costs must be discounted. For this 20-yr synthesis, carbon emissions and removals

were discounted to 2001 (start of study) with an annual discount rate of 4% (Searchinger et al. 2018) to get the "start-year-equivalent" costs. Under this approach, per-unit carbon emitted or removed in year 1 has a 4% higher absolute value than per-unit carbon emitted or removed in year 2.

S3.11 Stable carbon

The stable carbon pool is defined as the aboveground live tree carbon stored in large, fire-resistant trees expected to survive a simulated wildfire. This includes surviving individuals of fire-resistant species with DBH ≥ 72.6cm. The potential mortality was estimated by P-mort (Section S8).

Section S4. Data collection

In each experimental unit, 20 permanent 0.04-ha circular plots were established on a 60-m grid initiated with a random starting point. Plot locations were restricted to a 10-ha core area in the center of each unit to avoid edge effects (Stephens and Moghaddas 2005). Plot centers were permanently marked with a metal post and witness trees were recorded to ensure precise relocation of the plots.

To quantify responses to the treatment regime, the sampling design prioritized pre- and post-treatment measurements. Specifically, comprehensive field inventories were conducted in all units in the summers of 2001, 2003, 2009, 2016, and 2020 (Table S3). In addition, immediately after the prescribed fire treatments, "update" inventories were conducted in 2010 (2nd entry), and 2017 (3rd entry). Forest carbon pools were organized for sampling and reporting into the following categories: aboveground live tree, aboveground standing dead tree (snag), aboveground understory, fine woody debris (1-h, 10-h, and 100-h timelag fuel classes), coarse woody debris (1000-h and greater timelag fuel classes), litter, duff, and soil organic carbon

(SOC) in the 0-15 cm layer of mineral soil. Outlined below are the sampling protocols for each carbon pool with modifications noted for specific inventories.

S4.1 Live tree

For the comprehensive inventories, all live trees ≥ 11.4 cm DBH in the 0.04-ha plot were tagged and identified to species. For live trees, DBH, total height, height to live crown base, and crown position (dominant, co-dominant, intermediate, and suppressed) were recorded. Smaller live trees (1.0 cm ≤ DBH <11.4 cm) were sampled in 0.004-ha subplots in 2001, 2003, 2009, and 2016. In 2001, 2003, and 2009, species, DBH, total height, and height to live crown base were recorded for all smaller live trees. In 2016, small trees were tallied by species within binned diameter classes of 1.0–2.5, 2.5–5.1, 5.1–7.6, 7.6–10.2, and 10.2–11.4 cm. Each tallied small tree was assigned a DBH within its bin range from a uniform distribution, and assigned a total height based on observed relationships between DBH and height for small trees in the 2001, 2003, and 2009 data. In 2020, sampling of smaller trees was expanded to the entire 0.04 ha plot; DBH and height were measured along with species identification. For the post-fire update inventories of 2010 and 2017, the status of the tagged live trees post-fire was assessed, and any changes were recorded. Trees not impacted by the fire were assigned the pre-treatment size given the one-year difference between pre- and post-treatment measurements.

S4.2 Snag

All snags ≥ 11.4 cm DBH were tagged, identified to species if possible, and measured for DBH and total height (2001, 2003, and 2009 inventories). In 2016, only snags ≥ 20.5 cm DBH were recorded but in addition to DBH and total height, decay state was assessed. In 2020, all snags ≥ 11.4 cm DBH were measured and assigned a decay class (United States Forest Service 2024). For the post-fire update inventories of 2010 and 2017, the status of snags post-fire was

assessed, and any changes were recorded. Snags not impacted by the fire were assigned the pre-treatment measurements.

S4.3 Understory

The 0.04-ha plot was searched to identify all shrubs and herbs (i.e., forbs and grasses) present. Percent cover was visually estimated by species. Cover estimates were binned into classes of <5%, 5–25%, and 25–100%. The bins were interpreted as central values of 2.5%, 15%, and 63%, respectively, and these percent-cover categories were used to estimate the total area of cover by each species on each plot. In the 2016 and later inventories, the average height of each shrub species present was estimated along with the cover class.

S4.4 Fine and coarse woody debris

Surface and ground fuels (coarse woody debris, fine woody debris, litter, and duff) were sampled along radial transects (11.3 m) at two random azimuths in each 0.4-ha plot using the line intercept method (Brown 1974). Fuel measurements were taken on the same schedule as the vegetation measurements. The 1-h (0–0.64 cm) and 10-h (0.64–2.54 cm) fuels were sampled from 0 to 2 m, 100 h (2.54–7.62 cm) fuels from 0 to 3 m, and 1000 h (>7.62 cm) and larger fuels from 0 to 11.3 m on each transect. Surface and ground fuels were assessed in all inventories using the same protocol.

S4.5 Duff and litter depth

Litter and duff depths were measured at 0.3 and 0.9 m on each fuel transect. Per convention, litter was defined as the Oi fraction of the forest floor; duff included the Oe and Oa fractions (Brown 1974).

S4.6 Soil

Soil samples were collected in 2001, 2003, 2016, and 2020 with one exception. The most recent soil samples in the Mech units were collected in 2017 after the 2nd mastication application but before the understory thinning. In 2001 and 2003, six soil samples were obtained per plot. Litter and duff were collected in a 15-cm by 15-cm rectangular block; mineral soil to 15 cm depth was collected with a core (Moghaddas and Stephens 2007). Litter, duff, and mineral soil samples from each plot were aggregated prior to analysis. In 2016, two or three random samples were collected in each plot. Litter and duff were collected with a 30-cm diameter core; mineral soil to a 15-cm depth was collected with a 5-cm cylindrical core. Litter and duff were combined for processing. Each subsample (litter+duff and 0-15 cm mineral soil layer) was analyzed separately. In 2020 (2017 in the Mech), one sample was collected per plot. Litter and duff were collected in a 30-cm diameter core; two mineral soil cores (5-cm diameter) were collected at 0-15 cm depth and 15-30 cm depth. Litter and duff samples were combined for processing; the mineral soil samples were processed separately. In all cases, the depths of the litter layer and litter+duff were measured in the field.

Section S5. Carbon pools and costs estimation

S5.1 Aboveground live tree

Aboveground live tree biomass was calculated from tree measurements (species, DBH, and height) using regional biomass equations (Forest Inventory and Analysis 2010). These equations predict the biomass of the entire tree stem from estimates of cubic volume and species-specific wood density. Separate allometric equations were used to calculate the biomass in bark and branches. Foliage biomass was then calculated using Jenkins' ratios (Jenkins et al. 2003).

Aboveground live tree biomass is the sum of the stem, bark, branch, and foliage mass. The biomass estimate was converted to carbon using a carbon:biomass ratio of 0.48 (IPCC 2003).

S5.2 Snag

Snag biomass was initially estimated using the wood and bark equations for live trees described above and then corrected using a live:dead biomass ratio of 0.88. The 0.88 ratio was selected based on the findings of Cousins et al. (2015) for decay class 2, which was the modal and the median decay class for all snags in the 2016 inventory. The calculated biomass was converted to carbon using a carbon fraction of 0.5145 for snags in decay class 2 (Cousins et al. 2015; Foster et al. 2020). These estimates were summed and scaled by plot size to estimate the snag carbon pool (MgC/ha).

This simple discounting of snag carbon was necessary because decay class based on the FIA protocol (United States Forest Service 2024) was not systematically recorded for snags until 2016. However, for a subset of the snags in pre-treatment (2001) inventory, wildlife habitat assessments were available. These habitat assessments recorded variables (e.g., limb condition, wood hardness, and top presence) that were cross walked to the comparable FIA decay class (Foster et al. 2020). The modal decay class for snags in the pre-treatment plots was 2; it was also 2 for the snags in the 2016 inventory. For consistency across the two inventories, the same biomass decay and carbon fraction ratios were applied to all snags. Admittedly this assumption likely overestimates the biomass of old snags that have experienced significant degradation and decay. However, snag biomass is a relatively small pool and this bias does not influence the direction or magnitude of the cost of carbon analyses.

S5.3 Understory

Shrub biomass for each plot was calculated from biomass equations given by McGinnis et al. (2010). Using the crown area of the average-sized individuals for each species in McGinnes et al. (2010), shrub cover was converted into the estimated number of average-sized individuals present. The per-individual biomass was calculated using the species-specific allometric equations that predict total shrub biomass as a function of crown diameter and height. For earlier inventories with only cover, shrub height was predicted from the 2016 crown area and height data. These individual biomass estimates were summed and scaled by plot size to get shrub biomass on each plot (Mg/ha) and then converted to carbon (MgC/ha) using a carbon:biomass ration of 0.49 (Chojnacky and Milton 2008). The carbon density of herbs was calculated from cover using methods described in Campbell et al (2009). The understory carbon pool was estimated by summing the shrub and herb carbon pool.

S5.4 Fine and coarse woody debris

The biomass of woody debris was estimated from the transect data using equations and species-specific coefficients for Sierra Nevada forests (Van Wagdendonk, Benedict, and Sydoriak 1996; Van Wagtendonk, Benedict, and Sydoriak 1998). The coefficients used for each plot were generated by calculating the weighted mean of the species-specific coefficients with the weights derived from the relative basal area of the tree species present (Stephens 2001). The two transect-level estimates for woody debris biomass on each plot were averaged to generate a plot-level estimate, which was converted to Mg C/ha by assuming a carbon:biomass ratio of 0.5 for coarse and fine woody debris (IPCC 2003; Stephens et al. 2012).

S5.5 Litter and duff

Litter and duff samples were oven-dried at 65 °C to a constant weight and then ground through a 1-mm sieve. A 10-g sample of the 1-mm fraction was ground in a ball mill to pass a 60-mesh screen and then analyzed for total carbon by combustion (Moghaddas and Stephens 2007). Results from the 2016 analyses were used to quantify the consistency in the carbon fraction in litter and duff as estimated by depth. At the plot-level, litter and duff depths were reliable predictors of biomass ($R^2 = 0.85$, N = 267). Moreover, the carbon fraction of litter and duff samples demonstrated very little variability (Table S4). Therefore, to estimate carbon in the litter and duff pools, the depth measurements taken in the fuel transects were used to calculate biomass density based on methods outlined in Moghaddas and Stephens (2007). Litter biomass density was converted to carbon density using a carbon:biomass ratio of 0.463; for duff, biomass was converted to carbon using a ratio of 0.362 (Table S4).

S5.6 Mineral soil carbon

To determine the carbon content in the mineral soil, air-dry soil samples were sieved to obtain the fine fraction (particle size < 2 mm in diameter). A subsample of the fine fraction was dried to constant mass at 105 °C. A 10-g sample of the fine fraction was ground in a ball mill to pass a 60-mesh screen and then analyzed for total carbon by combustion (Moghaddas and Stephens 2007). Soil bulk density (g/cm^3) was determined based on the fine-fraction mass and total volume of each soil core. The bulk density of each sample and the carbon content of the fine fraction were used to determine the carbon pool in the surface soil on a per-hectare basis (MgC/ha).

S5.7 Fire emission and wildfire simulation

The First-Order Fire Effects Model (FOFEM 6.7, Keane and Lutes 2018) was applied to estimate the atmospheric carbon emissions (e.g., CO_2, CO, and CH_4) from both the prescribed fire (using Rx emission factors) and the simulated wildfire (using WF emission factor). The total emitted carbon was calculated based on the mass percentage of carbon compounds in the emission estimates. Inputs required for FOFEM modeling include plot-level pre-fire fuel loads, fuel moisture (duff, 10-hour, and 1000-hour fuels), understory biomass, crown biomass, and estimated crown burn percentage. For the prescribed fires, FOFEM was parameterized with the prevailing weather condition at the time of the burn, the measured pre-fire fuel loads, and the measured post-fire tree mortality. For the simulated wildfires, FOFEM used the weather conditions in the wildfire simulation, the measured 2020 (pre-wildfire) fuel loads, and the simulated post-fire tree mortality (Kennedy et al. 2020). Recent research at Blodgett Forest documented the skill of FOFEM in predicting emissions with measured pre-fire fuel loads (Tasnia et al. 2025).

S5.8 Wood product carbon

Harvested wood products (HWPs) are defined as all the woody material (including bark) that leaves the harvest site (IPCC 2006). HWP can be regarded as a carbon reservoir, but the time carbon is held in products varies depending on the product types (Finkral and Evans 2008). The wood products derived from harvested trees depend on the size and species. Most of the harvested wood from Blodgett Forest goes into long-lived wood products (LLP) with a small fraction used for fuelwood. Specifically, Douglas-fir and white fir are processed into dimensional lumber. Incense-cedar provides non-structural panels like fence boards and decking; ponderosa

pine and sugar pine produce non-structural products like doors and windowpanes. Black oak and tanoak (*Notholithocarpus densiflorus*) are used for fuelwood.

Dimensional lumber and non-structural products produced by the harvested softwood sawlogs in this study met the standards for long-lived products (LLP, Peng et al. 2023; Skog 2008). Therefore, these products were counted as a stable carbon stock for the 2001-2020 period. Harvested hardwood sawlogs used for fuelwood were considered as an immediate emission in the year of harvest (Peng et al. 2023). Harvested stems too small for sawlogs (i.e. non-merchantable) were left on site and processed according to the treatment strategy (i.e., either masticated or masticated and burned).

Harvested trees were recorded with an update inventory after mechanical treatments in 2017 (Mech) and 2019 (Mech+Fire). To calculate the carbon stored in LLP or used for fuelwood, the volume of all harvested sawlogs was estimated with regional volume equations (Forest Inventory and Analysis 2010) based on DBH and height. Sawlog volume for LLP only included conifers larger than 22.9 cm DBH (from a 0.3-m stump to a 15.2-cm diameter top). Based on recent estimates of California mill efficiency, 67.6% of the sawlog volume contributed to LLP (Buchholz et al. 2021). To account for the fossil fuel emissions associated with the harvest and transport, 5% of carbon stored in LLP was deducted from this pool (Buchholz et al. 2021). The kerf and trimmings from the processing (i.e., 22.4% of sawlog volume) are typically used as energy feedstock and thus were counted as an emission (Buchholz et al. 2021). Sawlog volume for fuelwood includes hardwoods larger than 27.9 cm DBH (from 0.3-m stump to 20.3-cm diameter top, Forest Inventory and Analysis 2010). These volumes were converted to carbon using the same methods to estimate live tree carbon, namely biomass was estimated from stem

volume using species-specific wood densities and then multiplied by a carbon:biomass ratio of 0.48.

In the 1st Mech treatment (2001), the tops and limbs were left in the forest; in the 2nd entry, the tops and limbs were hauled to the landing and burned. Thus, carbon in tops and limbs was added to the downed woody debris pools in 2001 but was an emission in 2019.

S5.9 Carbon cost

The cost of per-unit carbon emission is equivalent to the cost of per-unit mitigation. The mitigation cost is expected to decline with time due to innovation in mitigation technologies. This expectation of declining costs is based on two assumptions. One, existing mitigation options will get cheaper (Pacala and Socolow 2004). For example, the cost of solar panels and electric vehicles has dropped sharply (Gillingham and Stock 2018). Two, the development of novel technologies will make carbon capture more efficient and therefore more cost-effective (Wilberforce et al. 2021). In addition, the discount rate explicitly assigns a higher value to earlier mitigation efforts. It is an explicit recognition of the cumulative impact of increasing emissions on global warming (Peng et al. 2023). This analysis followed previous studies (Searchinger et al. 2018) and applied a discount rate of 4% per year. The results were reported in 2001 units to reflect “start-year equivalent” costs.

The annual carbon cost of treatments was based on the difference in the annual flux between the Control and wildfire-mitigation treatments (Peng et al. 2023). Any reduction in annual flux compared to Control was considered an emission; any increase – a sequestration. Emissions or sequestrations were assigned to the year the treatment was applied. The timing of the treatments varied over the course of the study with measurements bracketing each intervention (i.e., before and after treatment). To calculate the annual differences in carbon flux,

measured changes in carbon storage between two inventories were linearly interpolated. For example, the observed increase in total measured carbon between 2003 and 2009 in the Control was allocated to annual increments by dividing by the time between inventories (6 years, Table S5). Note that in this framework, the fraction of sawlog carbon stored in LLP was counted as a sequestration (i.e., not a cost even though they were no longer on site). In summary, the annual, time-discounted carbon costs were calculated and then these annual values were summed across 19 years to obtain the total carbon cost. The process used to calculate carbon costs is presented in detail in Section S8.

Section S6. Analytical framework

This study takes advantage of a long-term experiment with a robust statistical design. It uses a stock-change approach to quantify carbon dynamics over time. There is great value in having repeated observations over 19 years to track stocks and fluxes. However, the longevity of the study does pose analytical challenges. A particular challenge is the fact that not every plot was measured in every inventory. Some plots were lost mid-study to disturbance; other plots were inadvertently skipped during an inventory. Using a different set of plots through time to calculate the carbon balance risks confounding treatment effects with sampling effects. To limit this risk, all the results were calculated from plots in the unit that were measured at every interval. Thus, the sample size reported in the results reflects the number of consistently sampled plots. Another challenge, as noted above (Section S4), is that data collection protocols changed over time to accommodate resource constraints or to improve assessments. To the extent possible, these inconsistencies were minimized by the procedures used to calculate carbon pools (Section S6). All results were summarized at the plot level. Statistical analyses were conducted in R version 4.4.0 (R Core Team 2024).

Generalized linear mixed-effects models (GLMM) were used to test the BACI null hypothesis that the observed impacts of the treatment were no different from the after-treatment trajectory of responses in the controls (Christie et al. 2019). Specifically, the GLMMs examined the interaction between time and treatment on the carbon balance. In all the models, Control was set as the baseline for comparison. Random effects of the units were always included unless it reported a "singular fit" warning – an indication that the random effect variance was near zero.

Section S7. Analysis of treatment effects

Continuous response variables (e.g., total measured carbon) were evaluated using a linear mixed-effect model with a Gaussian distribution. Response variables were transformed when necessary to meet parametric assumptions. For proportional response variables (e.g., the proportion of species-specific fire-resistant carbon, proportion of large tree carbon), the default was a GLMM with Beta distribution (Douma and Weedon 2019). To meet the distribution requirement in the package, proportions equal to 1 in the data records were replaced with 0.99999. Validation tests on zero-inflation and dispersion provided by DHARMa package for R (Hartig and Lohse 2022) were used to decide if including zero-inflation or dispersion terms was necessary. If a zero-inflation term or dispersion term was required, their structure was based on the model with the lowest AIC (Akaike information criterion) values. Final models passed all validation tests.

To test whether the live vegetation recovery and surface fuel recovery rate after each entry of fire in the Fire units were significantly different from the Control units, another series of linear mixed-effects models were applied. The treatment type (Fire or Control) was the fixed effect. Pre-fire live vegetation carbon and disturbance intensity (carbon change during the treatment period) were included as covariates. Unit was included as the random effect. Though

the final data contained outliers, these outliers were ecologically reasonable. Thus, they were not removed. Under such minor violations of assumptions, the results of linear mixed-effect models are found to be generally robust (Schielzeth et al. 2020).

All the linear mixed-effect models were fitted with the lme4 package (Bates et al. 2024), and all the generalized linear mixed-effect models were fitted with the glmmTMB package (Brooks et al. 2024). Statistical results were reported only for the final models. For each model, we defined significant effects as those with a probability of occurring by chance was less than 0.05.

Section S8. Analysis of simulated wildfire

The Forest Vegetation Simulator-Fire and Fuels Extension (FVS-FFE, Rebain 2015) was used to understand the carbon consequence of a severe wildfire after 20 years of treatments (see Section S2). Key fire hazard metrics obtained from the potential fire report included the probability of torching (P-torch) and potential mortality (P-mort). P-torch is a stand-level index that estimates the proportion of the stand where torching is likely to occur; P-mort estimates the proportion of tree basal area killed by the fire (Rebain 2015). Species and size specific estimates of the trees killed by the fire, simulated results provided in FVS-FFE mortality report, were used to estimate the wildfire impacts on live tree carbon including losses in large-tree carbon and wildfire-resistant carbon. The live tree carbon stored in large, fire-resistant individuals that survived wildfire is considered as stable carbon (Section S3.11).

Section S8. Example of carbon cost calculation

The estimation of carbon costs is based on the annualized differences in carbon storage between the treatment and control units. To clarify the calculation, a detailed example is provided for Mech+Fire (see Table S5). In this example, **Year** is the calendar year; **Treatment scenario** is the mean carbon stored in the treated units for the given year; and **Control scenario** is the mean carbon stored in the control units for the given year. These carbon storage terms are the sum of the total measured carbon in the forest (Section S3.1) and the LLP pools stored outside the forest (Section S3.8). The **Control-Treatment** column is the difference in stored carbon between the two scenarios for the given year. Note that for 2001, the Control-Treatment column represents the pre-treatment differences in carbon storage between the units. Specifically, the value "-27.06" means that in 2001, the Mech+Fire units held 27.06 MgC/ha less carbon than the Control units. The **Undiscounted carbon cost** is calculated by subtracting the Control-Treatment value of the previous year from the entry of the current year. A positive value indicates the Treatment scenario resulted in a carbon emission relative to Control scenario; a negative value indicates a carbon sequestration. **Discount rate** is the compounded annual discount rate (4%) for carbon storage; **Carbon cost** of each year is the discounted cost for the given year. In this example, the undiscounted carbon cost during 2001–2020 is 145.88 MgC/ha and the carbon cost during 2001–2020 is 103.31 MgC/ha. To enable comparison among treatments, the carbon cost needs to be adjusted for the pre-treatment difference by subtracting it from the discounted carbon cost. Therefore, the final carbon cost for Mech+Fire equals: 103.31 MgC/ha – (-27.06 MgC/ha) = 130.4 MgC/ha (as reported in Table 4).

Table S1. Species composition measured as relative dominance (%) in the experimental treatments at Blodgett Forest Research Station. Reported values are means for the plots in each treatment. N_plot is the number of plots present in both 2001 and 2020.

Species	Control			Fire			Mech			Mech+Fire		
	**2001**	**2020**	**Difference**	**2001**	**2020**	**Difference**	**2001**	**2020**	**Difference**	**2001**	**2020**	**Difference**
**White fir**	22.6	21.0	-1.6	27.4	27.5	0.1	25.0	21.2	-3.8	18.7	13.1	-5.6
**Incense-cedar**	28.6	29.4	0.8	25.0	24.2	-0.8	21.5	20.5	-1.0	17.3	16.3	-1.0
**Sugar pine**	4.8	5.6	0.8	11.6	9.2	-2.4	13.6	15.4	1.8	14.5	17.0	2.4
**Ponderosa pine**	17.8	15.3	-2.5	12.7	9.9	-2.8	5.5	7.2	1.6	24.7	37.0	12.4
**Douglas-fir**	15.0	18.1	3.2	20.6	20.3	-0.4	24.2	26.8	2.6	12.2	12.6	0.4
**Black oak**	9.8	9.4	-0.5	2.1	5.2	3.1	9.1	7.4	-1.7	12.5	3.7	-8.9
N_plot	53			56			46			48		

Table S2. Basal area (m^2/ha) of live trees with a DBH > = 11.4cm in the experimental treatments at Blodgett Forest Research Station. Reported values are means and standard error (SE) from the plots in each treatment. N_plot is the number of plots present in both 2001 and 2020.

	**Control**				**Fire**				**Mech**				**Mech+Fire**			
	**Pre (2001)**		**Post (2020)**		**Pre (2001)**		**Post (2020)**		**Pre (2001)**		**Post (2020)**		**Pre (2001)**		**Post (2020)**	
	Mean	SE	Mean	SE	Mean	SE	Mean	SE	Mean	SE	Mean	SE	Mean	SE	Mean	SE
**Basal area**	54.7	2.7	68.2	3.2	48.5	2.0	49.7	2.4	51.6	2.0	43.8	2.3	55.3	2.5	34.5	2.4
N_plot	53				56				46				48			

Table S3. Timeline of measurements and treatments from 2001 to 2020 for the Fire and Fire Surrogate Study at Blodgett Forest Research Station.

**Treatments**	**1st entry**			**2nd entry**			**3rd entry**		
	**Pre**	**Update**	**Post**	**Pre**	**Update**	**Post**	**Pre**	**Update**	**Post**
**Control**	2001		2003	2009			2016		2020
**Fire**	2001		2003	2009	2010		2016	2017	2020
**Mech**	2001		2003	2016		2020			
**Mech+Fire**	2001		2003	2016		2020			

Table S4. Carbon fraction (% of dry biomass) of litter and duff samples from 2016 sampling at Blodgett Forest Research Station. SE = standard error of the mean; N = number of samples.

Fraction	N	Mean	SE
**Litter**	82	46.3	0.3
**Duff**	71	36.2	1.1
**Litter+Duff**	588	38.4	0.2

Table S5. Details on the calculation of the carbon cost of wildfire mitigation treatments. Treatment scenario below is based on the Mech+Fire treatment at Blodgett Forest Research Station. The carbon storage values in the Treatment and Control scenarios are plot means, N=40 for Treatment; N = 51 for Control).

Year	Treatment scenario (MgC/ha)	Control scenario (MgC/ha)	Control-Treatment (MgC/ha)	Undiscounted cost (MgC/ha)	Discount rate (%)	Carbon cost (MgC/ha)
2001	307.46	280.40	-27.06	**-27.06**	1.00	-27.06
2002	227.88	286.43	58.56	85.62	1.04	82.32
2003	232.37	292.46	60.09	1.53	1.08	1.42
2004	235.83	294.20	58.37	-1.72	1.12	-1.53
2005	239.30	295.95	56.65	-1.72	1.17	-1.47
2006	242.76	297.69	54.93	-1.72	1.22	-1.41
2007	246.22	299.44	53.22	-1.72	1.27	-1.36
2008	249.69	301.18	51.50	-1.72	1.32	-1.31
2009	253.15	302.93	49.78	-1.72	1.37	-1.26
2010	258.80	310.68	51.88	2.10	1.42	1.47
2011	264.45	318.42	53.97	2.10	1.48	1.42
2012	270.10	326.17	56.07	2.10	1.54	1.36
2013	275.75	333.92	58.17	2.10	1.60	1.31
2014	281.40	341.67	60.27	2.10	1.67	1.26
2015	287.05	349.41	62.36	2.10	1.73	1.21
2016	292.70	357.16	64.46	2.10	1.80	1.16
2017	178.88	365.95	187.06	122.60	1.87	65.46
2018	200.04	374.73	174.69	-12.37	1.95	-6.35
2019	191.52	383.52	192.00	17.31	2.03	8.54
2020	246.42	392.30	145.88	-46.12	2.11	-21.89
			**Sum**	**145.88**	-	**103.31**

Supplementary results

Table S6. Change in carbon storage (MgC/ha) by pool for the Fire and Fire Surrogate Study at Blodgett Forest Research Station.

Reported values are means and standard error (SE) from the plots in each treatment.

Carbon pool	Control				Fire				Mech				Mech+Fire			
	Pre (2001)		Post (2020)		Pre (2001)		Post (2020)		Pre (2001)		Post (2020)		Pre (2001)		Post (2020)	
	Mean	SE	Mean	SE	Mean	SE	Mean	SE	Mean	SE	Mean	SE	Mean	SE	Mean	SE
Aboveground live tree	171.6	10.3	245.0	14.7	149.8	8.4	186.3	10.7	169.9	8.6	171.8	11.7	188.5	11.4	133.9	9.9
Snag	4.5	1.6	10.3	2.1	6.3	2.6	7.1	1.6	10.2	2.5	7.2	2.2	6.1	1.5	21.8	5.9
Shrub	0.8	0.1	0.5	0.1	0.6	0.1	0.5	0.1	1.2	0.2	0.2	0.1	0.6	0.1	0.6	0.1
Find woody debris	5.7	0.6	6.2	0.7	6.0	0.5	3.7	0.3	4.8	0.4	6.8	0.6	6.0	0.5	3.0	0.3
Coarse woody debris	11.4	1.9	10.9	2.4	15.2	2.5	10.1	2.5	15.4	3.3	7.1	1.5	13.7	1.9	5.3	1.3
Duff	18.6	2.5	22.3	1.9	17.5	2.1	4.0	0.5	17.6	1.9	18.0	2.0	22.8	1.8	5.5	1.4
Litter	9.1	0.6	24.1	3.1	8.7	0.6	10.2	1.0	9.0	0.7	19.1	3.3	9.7	0.5	7.7	0.8
Mineral soil (0-15 cm)	58.7	1.2	71.2	2.6	57.6	1.3	60.8	2.1	57.5	1.4	53.2	1.9	57.8	1.5	56.8	2.4
**Total measured carbon**	280.4	11.1	390.5	15.2	261.7	10.5	282.8	10.6	285.5	10.1	283.4	11.6	305.2	12.0	234.5	10.6
Total number of plots	53				56				46				48			

Table S7. Linear mixed effect model results of the impact of wildfire-mitigation regimes on the carbon stocks.

	**Response variable: Sqrt(total measured carbon)**		
Predictors	*Estimates*	*CI*	*p*
(Intercept)	16.57	15.81 – 17.33	**<0.001**
Treatment:Fire	-0.57	-1.63 – 0.50	0.297
Treatment:Mech	0.20	-0.91 – 1.31	0.719
Treatment:Mech+Fire	0.74	-0.36 – 1.84	0.184
Treatment:Control x Time:2020	3.01	2.11 – 3.91	**<0.001**
Treatment:Fire x Time:2020	-2.36	-3.61 – -1.10	**<0.001**
Treatment:Mech x Time:2020	-3.11	-4.43 – -1.78	**<0.001**
Treatment:Mech+Fire x Time:2020	-5.20	-6.51 – -3.89	**<0.001**
**Random Effects**			
σ^2	5.58		
$\tau_{00\ comp}$	0.13		
ICC	0.02		
N_{comp}	12		
Observations	406		
Marginal R^2 / Conditional R^2	0.205 / 0.223		

Note: The response variable is the square-rooted total measured carbon (Section S3.1). “X” indicates the interaction term.

Table S8. Linear mixed effect model results of the post-fire live vegetation carbon recovery rate.

	1st Fire Recovery			2nd Fire Recovery			3rd Fire Recovery		
Predictors	*Estimates*	*CI*	*p*	*Estimates*	*CI*	*p*	*Estimates*	*CI*	*p*
(Intercept)	3.30	1.50 – 5.10	**<0.001**	-0.22	-2.17 – 1.73	0.822	-3.33	-7.26 – 0.61	0.097
Treatment:Fire	-3.06	-4.68 – -1.44	**<0.001**	0.56	-1.50 – 2.63	0.591	5.86	2.14 – 9.59	**0.002**
Pre-treatment live vegetation carbon	0.01	-0.00 – 0.02	0.057	0.03	0.02 – 0.03	**<0.001**	0.02	0.01 – 0.04	**0.007**
Disturbance intensity	-0.05	-0.18 – 0.08	0.435	-0.03	-0.08 – 0.01	0.177	-0.11	-0.39 – 0.18	0.458
**Random Effects**									
σ^2	10.18			7.43					
τ_{00}	0.40 $_{comp}$			1.13 $_{comp}$					
ICC	0.04			0.13					
N	6 $_{comp}$			6 $_{comp}$					
Observations	112			113			102		
Marginal R^2 / Conditional R^2	0.204 / 0.234			0.346 / 0.433					
R^2 / R^2 adjusted							0.202 / 0.177		

Note: Model for the 1st and 2nd fire recovery included random effect, model for the 3rd fire recovery did not include random effect.

Table S9. Linear mixed effect model results of the post-fire surface fuel carbon recovery rate.

	1st Fire Recovery			2nd Fire Recovery			3rd Fire Recovery		
Predictors	*Estimates*	*CI*	*p*	*Estimates*	*CI*	*p*	*Estimates*	*CI*	*p*
(Intercept)	1.85	1.31 – 2.38	**<0.001**	2.43	1.77 – 3.10	**<0.001**	6.40	3.68 – 9.13	**<0.001**
Treatment:Fire	-0.50	-1.01 – 0.01	0.053	-0.38	-0.93 – 0.16	0.165	-3.53	-5.76 – -1.31	**0.002**
Pre-treatment surface fuel carbon	-0.15	-0.19 – -0.12	**<0.001**	-0.14	-0.19 – -0.09	**<0.001**	-0.18	-0.31 – -0.04	**0.012**
Disturbance intensity	-0.16	-0.19 – -0.12	**<0.001**	-0.09	-0.17 – -0.02	**0.018**	-0.27	-0.44 – -0.09	**0.003**
**Random Effects**									
σ^2	0.70								
τ_{00}	0.00_{comp}								
ICC	0.00								
N	6_{comp}								
Observations	112			113			101		
Marginal R^2 / Conditional R^2	0.573 / 0.574								
R^2 / R^2 adjusted				0.234 / 0.213			0.125 / 0.098		

Note: Model for the 1st fire recovery included random effect, model for the 2nd and 3rd fire recovery did not include random effect.

Table S10. Generalized linear mixed effect model (GLMM) with beta distribution results of the impact of wildfire-mitigation regimes on large live tree carbon percentage (%).

	**Response variable: Large live tree carbon percentage**		
Predictors	*Estimates*	*CI*	*p*
**Count Model**			
(Intercept)	0.84	0.72 – 0.97	**0.022**
Treatment:Fire	0.95	0.78 – 1.16	0.646
Treatment:Mech	1.04	0.86 – 1.27	0.678
Treatment:Mech+Fire	1.20	0.97 – 1.49	0.099
Treatment:Control x Time:2020	1.28	1.04 – 1.57	**0.017**
Treatment:Fire x Time:2020	1.23	0.93 – 1.62	0.154
Treatment:Mech x Time:2020	1.46	1.10 – 1.94	**0.009**
Treatment:Mech+Fire x Time:2020	1.55	1.08 – 2.22	**0.016**
**Dispersion**			
(Intercept)	7.83	4.91 – 12.49	**<0.001**
Treatment:Fire	0.77	0.42 – 1.41	0.394
Treatment:Mech	1.42	0.73 – 2.73	0.299
Treatment:Mech+Fire	1.31	0.69 – 2.51	0.409
Treatment:Control x Time:2020	0.79	0.42 – 1.47	0.461
Treatment:Fire x Time:2020	0.56	0.24 – 1.30	0.174
Treatment:Mech x Time:2020	0.57	0.24 – 1.32	0.189
Treatment:Mech+Fire x Time:2020	0.23	0.10 – 0.54	**0.001**
**Zero-Inflated Model**			
(Intercept)	0.56	0.43 – 0.74	**<0.001**
Time:2020	0.36	0.23 – 0.56	**<0.001**
**Random Effects**			
σ^2	NA		
$\tau_{00\ comp}$	0.00		
N_{comp}	12		
Observations	438		

Note: “X” indicates the interaction term.

Table S11. Generalized linear mixed effect model (GLMM) with beta distribution results of the impact of wildfire-mitigation regimes on species-specific fire-resistant carbon percentage (%)

	Response variable: Species-specific fire resistant carbon percentage		
Predictors	*Estimates*	*CI*	*p*
**Count Model**			
(Intercept)	0.527	0.359 – 0.773	**0.001**
Treatment:Fire	1.078	0.626 – 1.858	0.786
Treatment:Mech	1.179	0.670 – 2.075	0.568
Treatment:Mech+Fire	1.611	0.959 – 2.708	0.072
Treatment:Control x Time:2020	0.700	0.438 – 1.119	0.136
Treatment:Fire x Time:2020	1.438	0.737 – 2.807	0.287
Treatment:Mech x Time:2020	1.310	0.635 – 2.701	0.465
Treatment:Mech+Fire x Time:2020	5.342	2.778 – 10.274	**<0.001**
**Dispersion**			
(Intercept)	8.205	6.229 – 11.264	
**Zero-Inflated Model**			
(Intercept)	0.325	0.211 – 0.502	**<0.001**
Treatment:Fire	1.404	0.784 – 2.515	0.254
Treatment:Mech	2.066	1.148 – 3.718	**0.015**
Treatment:Mech+Fire	0.535	0.269 – 1.061	0.074
**Random Effects**			
σ^2	0.00		
$\tau_{00\ comp}$	0.02		
ICC	1.00		
N_{comp}	12		
Observations	438		
Marginal R^2 / Conditional R^2	0.938 / 1.000		

Note: "X" indicates the interaction term.

Table S12. Trends in annual tree mortality at Blodgett Forest Research Station. Annual mortality calculated using the discrete rate (Sheil, Burslem, and Alder 1995). The 95% confidence interval (CI95) was estimated by resampling the binomial distribution. Harvested trees were removed from the analysis. N_0 is the number of trees at the start of the interval.

**Time**	**Treatment**	**N_0**	**Annual Mortality (%/year)**	
			Mean	CI95
**2003-2009**				
	Control	1,171	0.9	0.9 - 1.5
	Fire	936	4.7	4.7 - 6.1
	Mech	455	0.6	0.7 - 1.5
	Mech+Fire	396	3.5	3.5 - 5.3
**2010-2016**				
	Control	1,103	2.4	2.4 - 3.3
	Fire	670	3.1	3.0 - 4.5
	Mech	443	1.6	1.6 - 2.7
	Mech+Fire	315	2.0	1.9 - 3.8
**2017-2020**				
	Control	1,068	3.2	3.2 - 4.2
	Fire	578	2.7	2.6 - 4.2
	Mech	405	5.4	5.4 - 7.7
	Mech+Fire	267	6.3	6.4 - 9.4

Table S13. Median surface fuel accumulation or recovery rates (MgC/ha/yr) in Control and Fire treatments at Blodgett Forest Research Station. Results presented for each recovery interval.

Period	Control	Fire
**1st Fire Recovery** (2003-2009)	-0.17	0.82
**2nd Fire Recovery** (2010-2016)	0.81	0.68
**3rd Fire Recovery** (2017-2020)	1.39	1.53

Table S14. Wildfire-resistant carbon of different treatments in 2001 (before treatment), 2020 (after 20-yr treatments), and 2020 with simulated wildfire at Blodgett Forest Research Station. N =number of plots. Large trees refer to trees with DBH larger than 72.6cm; fire-resistant species include sugar pine and ponderosa pine (DBH ≥ 11.4 cm); Large fire-resistant tree includes that trees of fire-resistant species with DBH ≥ 72.6cm.

		**Large tree carbon (MgC/ha)**						**Fire-resistant species carbon (MgC/ha)**						**Large fire-resistant tree carbon (MgC/ha)**					
		Pre (2001)		Post (2020)		Post-wildfire (2020)		Pre (2001)		Post (2020)		Post-wildfire (2020)		Pre (2001)		Post (2020)		Post-wildfire (2020)	
Treatment	N	Mean	SE	Mean	SE	Mean	SE	Mean	SE	Mean	SE	Mean	SE	Mean	SE	Mean	SE	Mean	SE
Control	53	54.1	9.3	123.6	15.2	102.3	11.2	46.3	6.6	70.1	10.5	62.5	10.2	29.9	5.8	60.7	9.5	43.8	8.9
Fire	56	42.7	6.8	107.8	11.3	86.3	15.3	41.5	6.9	64.3	10.4	48.9	9.7	24.3	6.1	53.6	9.9	52.5	9.7
Mech	46	63.9	9.0	126.6	13.3	122.8	13.1	51.2	9.5	64.4	13.0	63.1	12.8	38.9	8.3	61.5	12.8	60.3	12.6
Mech+Fire	48	81.6	11.9	90.8	10.4	88.3	10.1	87.5	9.1	83.9	9.9	81.7	9.7	59.4	9.2	71.5	9.9	69.9	9.7

Table S15. Fate of materials harvested in the Mech and Mech+Fire treatments at Blodget Forest Research Station. Results reported by category in MgC/ha. N_plot=number of plots present in both 2002 and 2018.

Treatment	Time	N_plot	Left in field	Burned at landing	Bark	Fuelwood	Wood product (LLP)	Mill emission	Total
Mech	2002	46	10.4	0.0	0.0	1.4	6.7	3.2	21.7
Mech	2018	46	1.7	6.6	6.2	1.2	11.3	5.4	32.3
Mech+Fire	2002	48	11.4	0.0	0.0	1.9	6.6	3.2	23.1
Mech+Fire	2018	48	0.2	2.3	1.9	0.0	4.0	1.9	10.4

References:

Agee, James K., and Carl N. Skinner. 2005. “Basic Principles of Forest Fuel Reduction Treatments.” *Forest Ecology and Management*, Relative Risk Assessments for Decision –Making Related To Uncharacteristic Wildfire, 211 (1): 83–96. https://doi.org/10.1016/j.foreco.2005.01.034.

Bates, Douglas, Martin Maechler, Ben Bolker, Steven Walker, Rune Haubo Bojesen Christensen, Henrik Singmann, Bin Dai, et al. 2024. “Lme4: Linear Mixed-Effects Models Using ‘Eigen’ and S4.” https://cran.r-project.org/web/packages/lme4/index.html.

Brooks, Mollie, Ben Bolker, Kasper Kristensen, Martin Maechler, Arni Magnusson, Maeve McGillycuddy, Hans Skaug, et al. 2024. “glmmTMB: Generalized Linear Mixed Models Using Template Model Builder.” https://cran.r-project.org/web/packages/glmmTMB/index.html.

Brown, James K. 1974. “Handbook for Inventorying Downed Woody Material.”

Buchholz, Thomas, Tad Mason, Bruce Springsteen, John Gunn, and David Saah. 2021. “Carbon Life Cycle Assessment on California-Specific Wood Products Industries: Do Data Backup General Default Values for Wood Harvest and Processing?” *Forests* 12 (2): 177. https://doi.org/10.3390/f12020177.

Campbell, John L., Giorgio Alberti, Jonathan Martin, and Beverly E. Law. 2009. “Carbon Dynamics of a Ponderosa Pine Plantation Following a Thinning Treatment in the Northern Sierra Nevada.” *Forest Ecology and Management* 257 (2): 453–63. https://doi.org/10.1016/j.foreco.2008.09.021.

Cansler, C. Alina, Sharon M. Hood, Phillip J. van Mantgem, and J. Morgan Varner. 2020. “A Large Database Supports the Use of Simple Models of Post-Fire Tree Mortality for Thick-Barked Conifers, with Less Support for Other Species.” *Fire Ecology* 16 (1): 25. https://doi.org/10.1186/s42408-020-00082-0.

Chojnacky, David C., and Mikaila Milton. 2008. “Measuring Carbon in Shrubs.” In *Field Measurements for Forest Carbon Monitoring: A Landscape-Scale Approach*, edited by Coeli M. Hoover, 45–72. Dordrecht: Springer Netherlands. https://doi.org/10.1007/978-1-4020-8506-2_5.

Christie, Alec P., Tatsuya Amano, Philip A. Martin, Gorm E. Shackelford, Benno I. Simmons, and William J. Sutherland. 2019. "Simple Study Designs in Ecology Produce Inaccurate Estimates of Biodiversity Responses." *Journal of Applied Ecology* 56 (12): 2742–54. https://doi.org/10.1111/1365-2664.13499.

Cousins, Stella J. M., John J. Battles, John E. Sanders, and Robert A. York. 2015. "Decay Patterns and Carbon Density of Standing Dead Trees in California Mixed Conifer Forests." *Forest Ecology and Management* 353 (1): 136–47. https://doi.org/10.1016/j.foreco.2015.05.030.

Daniel, Kent D., Robert B. Litterman, and Gernot Wagner. 2019. "Declining CO2 Price Paths." *Proceedings of the National Academy of Sciences* 116 (42): 20886–91. https://doi.org/10.1073/pnas.1817444116.

Douma, Jacob C., and James T. Weedon. 2019. "Analysing Continuous Proportions in Ecology and Evolution: A Practical Introduction to Beta and Dirichlet Regression." *Methods in Ecology and Evolution* 10 (9): 1412–30. https://doi.org/10.1111/2041-210X.13234.

Finkral, Alex J., and Alexander M. Evans. 2008. "The Effects of a Thinning Treatment on Carbon Stocks in a Northern Arizona Ponderosa Pine Forest." *Forest Ecology and Management*, Large-scale experimentation and oak regeneration, 255 (7): 2743–50. https://doi.org/10.1016/j.foreco.2008.01.041.

Forest Inventory and Analysis. 2010. *Regional Biomass Equations Used by the Forest Inventory and Analysis Program to Estimate Bole, Bark, and Branches (Updated 13-Jan-2010)*. Portland, Oregon, USA: USDA Forest Service.

Foster, Daniel E., John J. Battles, Brandon M. Collins, Robert A. York, and Scott L. Stephens. 2020. "Potential Wildfire and Carbon Stability in Frequent-Fire Forests in the Sierra Nevada: Trade-Offs from a Long-Term Study." *Ecosphere* 11 (8): e03198. https://doi.org/10.1002/ecs2.3198.

Graham, Russell T., Sarah McCaffrey, and Theresa B. Jain. 2004. "Science Basis for Changing Forest Structure to Modify Wildfire Behavior and Severity." RMRS-GTR-120. Ft. Collins, CO: U.S.

Department of Agriculture, Forest Service, Rocky Mountain Research Station. https://doi.org/10.2737/RMRS-GTR-120.

Guadalupe, Lara. 2022. "Application and Evaluation of FOFEM in a Mixed Conifer Forest at the Blodgett Forest." UC Riverside. https://escholarship.org/uc/item/4r9256x9.

Hartig, Florian, and Lukas Lohse. 2022. "DHARMa: Residual Diagnostics for Hierarchical (Multi-Level / Mixed) Regression Models." https://cran.r-project.org/web/packages/DHARMa/index.html.

IPCC. 2003. "Good Practice Guidance for Land Use, Land-Use Change and Forestry." Kanagawa: Institute for Global Environmental Strategies (IGES).

———. 2006. "Guidelines for National Greenhouse Gas Inventories." IGES, Japan: National Greenhouse Gas Inventories Programme. https://www.ipcc.ch/report/2006-ipcc-guidelines-for-national-greenhouse-gas-inventories/.

Jenkins, Jennifer C., David C. Chojnacky, Linda S. Heath, and Richard A. Birdsey. 2003. "National Scale Biomass Estimators for United States Tree Species." *Forest Science. 49: 12-35*. https://research.fs.usda.gov/treesearch/6996.

McGinnis, Thomas W., Christine D. Shook, and Jon E. Keeley. 2010. "Estimating Aboveground Biomass for Broadleaf Woody Plants and Young Conifers in Sierra Nevada, California, Forests." *Western Journal of Applied Forestry* 25 (4): 203–9. https://doi.org/10.1093/wjaf/25.4.203.

McIver, James D., Scott L. Stephens, James K. Agee, Jamie Barbour, Ralph E. J. Boerner, Carl B. Edminster, Karen L. Erickson, et al. 2012. "Ecological Effects of Alternative Fuel-Reduction Treatments: Highlights of the National Fire and Fire Surrogate Study (FFS)." *International Journal of Wildland Fire* 22 (1): 63–82. https://doi.org/10.1071/WF11130.

Moghaddas, Emily E. Y., and Scott L. Stephens. 2007. "Thinning, Burning, and Thin-Burn Fuel Treatment Effects on Soil Properties in a Sierra Nevada Mixed-Conifer Forest." *Forest Ecology and Management* 250 (3): 156–66. https://doi.org/10.1016/j.foreco.2007.05.011.

Peng, Liqing, Timothy D. Searchinger, Jessica Zionts, and Richard Waite. 2023. "The Carbon Costs of Global Wood Harvests." *Nature* 620 (7972): 110–15. https://doi.org/10.1038/s41586-023-06187-1.

Rodman, Kyle C., Thomas T. Veblen, Teresa B. Chapman, Monica T. Rother, Andreas P. Wion, and Miranda D. Redmond. 2020. "Limitations to Recovery Following Wildfire in Dry Forests of Southern Colorado and Northern New Mexico, USA." *Ecological Applications* 30 (1): e02001. https://doi.org/10.1002/eap.2001.

Schielzeth, Holger, Niels J. Dingemanse, Shinichi Nakagawa, David F. Westneat, Hassen Allegue, Céline Teplitsky, Denis Réale, Ned A. Dochtermann, László Zsolt Garamszegi, and Yimen G. Araya-Ajoy. 2020. "Robustness of Linear Mixed-Effects Models to Violations of Distributional Assumptions." *Methods in Ecology and Evolution* 11 (9): 1141–52. https://doi.org/10.1111/2041-210X.13434.

Schwilk, Dylan W., Jon E. Keeley, Eric E. Knapp, James McIver, John D. Bailey, Christopher J. Fettig, Carl E. Fiedler, et al. 2009. "The National Fire and Fire Surrogate Study: Effects of Fuel Reduction Methods on Forest Vegetation Structure and Fuels." *Ecological Applications* 19 (2): 285–304. https://doi.org/10.1890/07-1747.1.

Searchinger, Timothy D., Stefan Wirsenius, Tim Beringer, and Patrice Dumas. 2018. "Assessing the Efficiency of Changes in Land Use for Mitigating Climate Change." *Nature* 564 (7735): 249–53. https://doi.org/10.1038/s41586-018-0757-z.

Sheil, Douglas, David F. R. P. Burslem, and Denis Alder. 1995. "The Interpretation and Misinterpretation of Mortality Rate Measures." *The Journal of Ecology* 83 (2): 331. https://doi.org/10.2307/2261571.

Skinner, Carl N. and Stephens, Scott L. In press. The national fire and fire surrogates study at twenty years. *Ecological Applications*.

Skog, Kenneth E. 2008. "Sequestration of Carbon in Harvested Wood Products for the United States." *Forest Products Journal* 58 (6): 56–72.

Stephens, Scott L. 2001. “Fire History Differences in Adjacent Jeffrey Pine and Upper Montane Forests in the Eastern Sierra Nevada.” *International Journal of Wildland Fire* 10 (2): 161–67. https://doi.org/10.1071/wf01008.

———. 2021. “Sierra Nevada Forest Restoration Works: A Summary of the Fire and Fire Surrogate Study,” September. https://escholarship.org/uc/item/7kp9n1s8.

Stephens, Scott L., Ralph E. J. Boerner, Jason J. Moghaddas, Emily E. Y. Moghaddas, Brandon M. Collins, Christopher B. Dow, Carl Edminster, et al. 2012. “Fuel Treatment Impacts on Estimated Wildfire Carbon Loss from Forests in Montana, Oregon, California, and Arizona.” *Ecosphere* 3 (5): 1–17. https://doi.org/10.1890/ES11-00289.1.

Stephens, Scott L., and Brandon M. Collins. 2004. “Fire Regimes of Mixed Conifer Forests in the North-Central Sierra Nevada at Multiple Spatial Scales.” *Northwest Science* 78 (1): 12–23.

Stephens, Scott L., Daniel E. Foster, John J. Battles, Alexis A. Bernal, Brandon M. Collins, Rachelle Hedges, Jason J. Moghaddas, Ariel T. Roughton, and Robert A. York. 2023. “Forest Restoration and Fuels Reduction Work: Different Pathways for Achieving Success in the Sierra Nevada.” *Ecological Applications*, e2932. https://doi.org/10.1002/eap.2932.

———. 2024. “Forest Restoration and Fuels Reduction Work: Different Pathways for Achieving Success in the Sierra Nevada.” *Ecological Applications* 34 (2): e2932. https://doi.org/10.1002/eap.2932.

Stephens, Scott L., and Jason J. Moghaddas. 2005. “Experimental Fuel Treatment Impacts on Forest Structure, Potential Fire Behavior, and Predicted Tree Mortality in a California Mixed Conifer Forest.” *Forest Ecology and Management* 215 (1): 21–36. https://doi.org/10.1016/j.foreco.2005.03.070.

Stephens, Scott L., Jason J. Moghaddas, Bruce R. Hartsough, Emily E.Y. Moghaddas, and Nicholas E. Clinton. 2009. “Fuel Treatment Effects on Stand-Level Carbon Pools, Treatment-Related Emissions, and Fire Risk in a Sierra Nevada Mixed-Conifer Forest.” *Canadian Journal of Forest Research* 39 (8): 1538–47. https://doi.org/10.1139/X09-081.

Stevens, Jens T., Matthew M. Kling, Dylan W. Schwilk, J. Morgan Varner, and Jeffrey M. Kane. 2020. "Biogeography of Fire Regimes in Western U.S. Conifer Forests: A Trait-based Approach." Edited by Thomas Gillespie. *Global Ecology and Biogeography* 29 (5): 944–55. https://doi.org/10.1111/geb.13079.

Stewart-Oaten, Allan, and James R. Bence. 2001. "Temporal and Spatial Variation in Environmental Impact Assessment." *Ecological Monographs* 71 (2): 305–39. https://doi.org/10.1890/0012-9615(2001)071[0305:TASVIE]2.0.CO;2.

Tasnia, A., Lara, G., Foster, D., Sengupta, D., Butler, J. D. A., Kirchstetter, T. W., York, R., Kreisberg, N. M., Goldstein, A. H., Battles, J. J., & Barsanti, K. C. 2025. Comprehensive Fuel and Emissions Measurements Highlight Uncertainties in Smoke Production Using Predictive Modeling Tools. ACS ES&T Air, 2(6), 982–997. https://doi.org/10.1021/acsestair.4c00142

United States Forest Service. 2024. "Forest Inventory and Analysis National Core Field Guide for the Nationwide Forest Inventory Version 9.4." https://research.fs.usda.gov/sites/default/files/2024-02/wo-v9-3_sep2023_fg_nfi_natl.pdf.

Van Mantgem, Phillip, and Mark Schwartz. 2003. "Bark Heat Resistance of Small Trees in Californian Mixed Conifer Forests: Testing Some Model Assumptions." *Forest Ecology and Management* 178 (3): 341–52. https://doi.org/10.1016/S0378-1127(02)00554-6.

Van Wagdendonk, Jan W., James M. Benedict, and Walter M. Sydoriak. 1996. "Physical Properties of Woody Fuel Particles of Sierra Nevada Conifers." *International Journal of Wildland Fire* 6 (3): 117–23. https://doi.org/10.1071/wf9960117.

Van Wagtendonk, Jan W., James M. Benedict, and Walter M. Sydoriak. 1998. "Fuel Bed Characteristics of Sierra Nevada Conifers." *Western Journal of Applied Forestry* 13 (3): 73–84. https://doi.org/10.1093/wjaf/13.3.73.

Wilberforce, Tabbi, A. G. Olabi, Enas Taha Sayed, Khaled Elsaid, and Mohammad Ali Abdelkareem. 2021. "Progress in Carbon Capture Technologies." *Science of The Total Environment* 761 (March):143203. https://doi.org/10.1016/j.scitotenv.2020.143203.

York, Robert A., Jacob Levine, Kane Russell, and Joseph Restaino. 2021. "Opportunities for Winter Prescribed Burning in Mixed Conifer Plantations of the Sierra Nevada." *Fire Ecology* 17 (1): 33. https://doi.org/10.1186/s42408-021-00120-5.
